# The global and regional costs of healthy and sustainable dietary patterns: a modelling study

**DOI:** 10.1016/S2542-5196(21)00251-5

**Published:** 2021-10-27

**Authors:** Marco Springmann, Michael A Clark, Mike Rayner, Peter Scarborough, Patrick Webb

**Affiliations:** a Oxford Martin Programme on the Future of Food and WHO Collaborating Centre on Population Approaches for Non-Communicable Disease Prevention, Nuffield Department of Population Health, University of Oxford, Oxford, UK; b NIHR Biomedical Research Centre, John Radcliffe Hospital, Oxford, UK; c Friedman School of Nutrition Science and Policy, Tufts University, Boston, MA, USA

## Abstract

**Background:**

Adoption of healthy and sustainable diets could be essential for safe-guarding the Earth's natural resources and reducing diet-related mortality, but their adoption could be hampered if such diets proved to be more expensive and unaffordable for some populations. Therefore, we aimed to estimate the costs of healthy and sustainable diets around the world.

**Methods:**

In this modelling study, we used regionally comparable food prices from the International Comparison Program for 150 countries. We paired those prices with estimates of food demand for different dietary patterns that, in modelling studies, have been associated with reductions in premature mortality and environmental resource demand, including nutritionally balanced flexitarian, pescatarian, vegetarian, and vegan diets. We used estimates of food waste and projections of food demand and prices to specify food system and socioeconomic change scenarios up to 2050. In the full cost accounting, we estimated diet-related health-care costs by pairing a comparative risk assessment of dietary risks with cost-of-illness estimates, and we estimated climate change costs by pairing the diet scenarios with greenhouse gas emission footprints and estimates of the social cost of carbon.

**Findings:**

Compared with the cost of current diets, the healthy and sustainable dietary patterns were, depending on the pattern, up to 22–34% lower in cost in upper-middle-income to high-income countries on average (when considering statistical means), but at least 18–29% more expensive in lower-middle-income to low-income countries. Reductions in food waste, a favourable socioeconomic development scenario, and a fuller cost accounting that included the diet-related costs of climate change and health care in the cost of diets increased the affordability of the dietary patterns in our future projections. When these measures were combined, the healthy and sustainable dietary patterns were up to 25–29% lower in cost in low-income to lower-middle-income countries, and up to 37% lower in cost on average, for the year 2050. Variants of vegetarian and vegan dietary patterns were generally most affordable, and pescatarian diets were least affordable.

**Interpretation:**

In high-income and upper-middle-income countries, dietary change interventions that incentivise adoption of healthy and sustainable diets can help consumers in those countries reduce costs while, at the same time, contribute to fulfilling national climate change commitments and reduce public health spending. In low-income and lower-middle-income countries, healthy and sustainable diets are substantially less costly than western diets and can also be cost-competitive in the medium-to-long term, subject to beneficial socioeconomic development and reductions in food waste. A fuller accounting of the costs of diets would make healthy and sustainable diets the least costly option in most countries in the future.

**Funding:**

Global Panel on Agriculture and Food Systems for Nutrition and Wellcome Trust.

## Introduction

The food system is facing increasing environmental and health challenges. At present, it is responsible for about a third of all greenhouse gas emissions,^1^ and without dedicated measures, its environmental impacts risk exceeding key environmental limits for climate change, land use, freshwater extraction, and biogeochemical flows associated with fertiliser application.^2^ At the same time, more than a quarter of deaths globally have been attributed to imbalanced diets,^3^ mostly from diet-related, chronic diseases that also require costly treatment.^4^, ^5^ Dietary changes towards nutritionally balanced diets that are low in animal products and high in nutritionally important plant-based foods—such as fruits, vegetables, legumes, nuts, and whole grains—have been proposed as an important measure to reduce the food system's growing environmental pressures, while improving nutritional status and dietary health.^2^, ^6^, ^7^

Although the importance and benefits of dietary changes towards healthy and sustainable diets are increasingly recognised,^5^, ^7^ much less is known about the economic dimensions of such changes, including the affordability and costs of diets. A global analysis of one particular healthy and sustainable dietary pattern found that it could be unaffordable for a large number of people in low-income countries,^8^ and a systematic review based on market and dietary surveys found that in high-income countries, healthier dietary patterns were on average more expensive than less healthy patterns.^9^ On the other hand, optimisation studies have shown that healthier and more sustainable diets can in principle be obtained without increases in costs in the countries that were analysed.^10^, ^11^, ^12^, ^13^ However, the results from these studies are difficult to generalise, because they either focused on one specific global diet, assessed diets that were not comparable across regions, or were limited to high-income countries, and collected price data in different ways in each case study. In addition, a focus on current market prices has little relevance for policy making in a context of changing food systems, economic conditions, and prices.^14^

**Research in context**

**Evidence before this study**

The existing evidence on the relative costs of healthy and sustainable diets is conflicting. A global analysis of one particular healthy and sustainable dietary pattern found that it could be unaffordable for many people in low-income countries. A systematic review based on market and dietary surveys found that in high-income countries, healthier dietary patterns were on average more expensive than less healthy patterns. Optimisation studies have shown that healthier and more sustainable diets can in principle be obtained without increases in costs in the countries that were analysed. The results from these studies are difficult to generalise because they either focused on one specific global diet, assessed diets that were not comparable across regions, or were limited to high-income countries, and they collected price data in different ways in each case study. In addition, a focus on current market prices has little relevance for policy making in a context of changing food systems, economic conditions, and prices.

**Added value of this study**

With our study, we advance the literature in three ways. First, we present regionally comparable estimates of diet costs for a standardised set of healthy and sustainable dietary patterns—including flexitarian, pescatarian, vegetarian, and vegan dietary patterns—for 150 countries, based on current food prices. Second, we assess the implications of food-system and socioeconomic changes on the cost of diets, including reductions in food waste at the household level and future changes in food prices and demand. Third, we value and include in our analysis two cost components that are currently not accounted for in the costs of diet or foods, in particular the costs of diet-related illness and the diet-related impacts on climate change. At present, these so-called external costs are levied onto society in ways other than through food prices, which distorts prices and can contribute to consumption decisions that are detrimental for public health and the environment.

**Implications of all the available evidence**

Several dietary patterns that, in modelled studies, have been assessed as healthier and more sustainable than current diets, can be lower in cost than current diets in most high-income and many middle-income countries, but higher in cost in low-income countries. Reductions in food waste and future socioeconomic changes can increase their relative affordability, especially in middle-income and some low-income countries, and a fuller cost accounting including the diet-related costs of climate change and health care in the cost of diets can further increase the relative affordability to all countries, including low-income countries. Across the dietary patterns, the relative affordability was largest for vegetarian and vegan diets that focused on legumes and whole grains in place of animal products in current diets, and lowest for pescatarian diets that focused on fish and fruits and vegetables.

In this Article, we advance the literature in three ways. First, we present regionally comparable estimates of diet costs for a standardised set of healthy and sustainable dietary patterns, including flexitarian, pescatarian, vegetarian, and vegan dietary patterns, for 150 countries using current food prices. Second, we assess the implications of food-system and socioeconomic changes on the cost of diets, including reductions in food waste at the household level, and future changes in food prices and demand. Third, we value and include in our analysis two cost components that are currently not accounted for in the costs of diets or foods, the costs of diet-related illness and the diet-related impacts on climate change. At present, these external costs are levied onto society in ways other than through food prices, which distorts prices and can contribute to consumption decisions that are detrimental for public health and the environment.^7^, ^15^, ^16^

## Methods

### Overview

In this modelling study, we calculated the costs of diets in 150 countries from all world regions by pairing estimates of food demand for different consumption patterns with estimates of commodity prices in different years and under considerations of food-system and socioeconomic changes.

### Price data

The price data were based on a detailed list of commodity prices collected by statistical offices for the year 2017 as part of the International Comparison Program led by the World Bank.^17^ For our analysis, we used 20 666 estimates of annual average prices in 179 countries, covering 463 food items (appendix 1 pp 2–16). We focused on those commodities that are related to foods, can be expressed in primary commodity equivalents, and are not beverages, except for milk. The items consisted of 319 items from regional lists, which are representative of the consumption pattern in the region, and 144 items from a global core list, which has been developed for the specific purpose of linking the regional results into a global set of results by including products that can be priced across most regions.

We aggregated the detailed list of food items into a list of 31 food groups that we used to construct the diet scenarios. For the aggregation, we paired each item with its caloric content (to control for difference in processing and edible fractions), and converted averaged prices from local currency to US$ (2017). For the calorie conversion, we used calorie data from the FoodData Central database maintained by the US Department of Agriculture. The price conversion was based on the application of purchasing-power-parity rates that control for differences in price levels across countries.

### Consumption data

To estimate current food consumption, we used globally comparable estimates of the amount of food that is available for consumption in a country, provided by the Food and Agriculture Organization of the UN, and we adjusted these estimates for food wasted during consumption and the difference between edible and inedible parts using region-specific and commodity-specific estimates (appendix 1 pp 17–19).^18^, ^19^ An alternative would have been to use estimates from dietary surveys, or household budget and expenditure surveys.^20^, ^21^ However, the available global data do not include enough food groups to represent complete diets, and the more complete survey data that are available for some individual countries are often subject to substantial underreporting and therefore not regionally comparable.^22^, ^23^ Waste-adjusted food availability estimates have their own caveats—eg, they do not explicitly include food from home production or differentiate subgroups within a population, some estimates are based on statistical imputation of data from previous years, and some aspects such as food stocks and waste would benefit from more regular updates—but in contrast to dietary surveys, they describe levels of energy intake that more accurately reflect the observed differences in the prevalence of overweight and obesity across regions,^24^ and accounting for the related level of overconsumption is an important aspect for estimating the cost of diets. The pairing of the consumption and price data resulted in a complete set of estimates for 150 countries.

### Diet scenarios

The healthy and sustainable diet scenarios included dietary patterns that, in modelling studies, have been associated with improvements in nutritional adequacy, and reductions in premature mortality and environmental resource demand.^2^, ^6^ In line with the sustainable-diet literature,^6^, ^7^, ^25^, ^26^, ^27^ we differentiated between four nutritionally balanced (ie, fulfilling nutrient requirements) and predominantly plant-based dietary patterns, in particular flexitarian, pescatarian (with increased seafood demand to be met by sustainable aquaculture), vegetarian, and vegan diets (appendix 1 pp 20–23). The flexitarian diet was adopted from the EAT-*Lancet* Commission on healthy diets from sustainable food systems.^7^ It included at least 500 g per day of fruits and vegetables, at least 100 g per day of plant-based protein sources (eg, legumes, soybeans, nuts), a focus on whole grains, modest amounts of animal-based proteins (eg, less than two servings per week of each poultry, fish, and eggs, and less than one serving per day of milk or dairy), and limited amounts of red meat (one portion per week or less), refined sugar (less than 5% of total energy), and vegetable oils that are high in saturated fat such as palm oil (less than one serving per day).

The more specialised dietary patterns were constructed by replacing the amount of animal products in the flexitarian diets. Because the exact composition of those diets is variable, we constructed two variants of each pattern, in which animal products were replaced by a mix of fish (pescatarian) or legumes (vegetarian, vegan) and either fruits and vegetables (high-veg variant) or whole grains (high-grain variant). The two variants of each specialised dietary pattern are meant to capture the diversity of such patterns and highlight particular trade-offs that are relevant for affordability as whole grains are usually cheaper per calorie than fruits and vegetables. The high-grain variants contained the same amount of fruits and vegetables as the flexitarian diets, and 2–9% more grains (by weight) than the high-veg variants (which still is about a third less than current diets; appendix 1 p 23).

Each scenario was regionalised by preserving each country's relative preferences for the types of grains, fruits, red meat, and fish, and by adjusting the intake of staple grains such that total energy intake was in line with the age-specific and sex-specific energy needs of a moderately active population in each country (appendix 1 pp 20–23).^28^, ^29^ Because we were interested in the costs of diets, and not only in the costs of final consumption, we added the commodity and region-specific estimates of food wasted at home to the estimates of food intake in each scenario,^18^ including the baseline estimates. We differentiated between a scenario in which food waste stays constant, and one in which it is halved, in line with the ambition expressed in the Sustainable Development Goals.^30^

### Price projections

To analyse the implications that different socioeconomic development trajectories could have for the relative differences in the cost of diets, we used price and demand projections from the International Model for Policy Analysis of Agricultural Commodities and Trade (IMPACT).^31^ IMPACT is a global partial equilibrium multimarket model of agricultural production, demand, trade, and prices. Its projections are in line with other agriculture-economic models,^32^ and they include future food demand and commodity prices for 62 agricultural commodities in over 150 countries and world regions. For our analysis, we used IMPACT's estimates of consumer prices and food demand for three different socioeconomic pathways that differed in their assumptions on income and population growth. They included a middle-of-the-road trajectory (SSP2), a more optimistic trajectory with higher income and lower population growth (SSP1), and a more pessimistic one with lower income and higher population growth (SSP3).^31^, ^33^ In each case, we calculated the percentage changes between the years of 2030 and 2050, and the year 2017, and applied those changes to our baseline estimates of food prices and demand (appendix 1 pp 24–26).

### External costs

External costs describe those costs that are associated with a specific good but are currently not accounted for in its price. Here we included two components that are related to diets and have previously been valued: the costs of climate change and the costs of illness.^5^ We followed existing methods, but updated the previous valuation in several ways. For the valuation of the diet-related costs of climate change, we first calculated diet-related greenhouse gas emissions by pairing the estimates of food demand in the different diet scenarios with footprints of greenhouse gas emissions from life-cycle assessments that were differentiated by commodity and region and accounted for future improvements in management and technology (appendix 1 pp 27–32).^34^, ^35^ We then paired those estimates with estimates of the social cost of carbon that represents the economic cost caused by an additional ton of greenhouse gas emissions. Compared with the earlier valuation, we used estimates from a fully revised version of the Dynamic Integrated model of Climate and the Economy for a scenario that constrains future temperature rise in line with stated policy goals.^36^

To estimate the health-care-related costs associated with the different diet scenarios, we used a comparative risk assessment framework with four disease endpoints (appendix 1 pp 33–34). Compared with the earlier valuation, the number of risk factors was extended from five to ten,^6^ and consisted of low intake of fruits, vegetables, legumes, nuts, and whole grains as well as high intake of red meat and processed meat, and three weight-related risks, namely being underweight, overweight, or obese. Relative risk estimates for each risk-disease association were adopted from meta-analyses of prospective cohort studies.^37^, ^38^, ^39^, ^40^, ^41^, ^42^, ^43^, ^44^, ^45^ For valuing the burden of dietary risks, we paired the cause-specific mortality estimates with an existing set of cost-of-illness estimates that were differentiated by country and disease endpoint.^5^ The dataset accounted for both the direct and indirect costs associated with treating a specific disease, including medical and health-care costs, and the costs of informal care and from lost working days. We estimated avoidable health-care costs in the different diet scenarios in relation to the scenario with the greatest reduction in diet-related disease mortality.

### Uncertainty analysis

We assessed the uncertainty of our estimates in several ways. For the price data, we used the range of prices for the food items on the global core list to calculate the mean values and 95% CIs for the more aggregated food groups. For the consumption and price projections, we used the spread of the different socioeconomic scenarios as uncertainty intervals. For the valuation of the health and climate change costs, we accounted for the uncertainty related to the relative risk factor in the health analysis, the spread of cost-of-illness estimates in the health valuation, the CIs of the emissions footprints in the environmental analysis, and different discount rates in the valuation of climate change costs.

### Data availability

The full set of results for all 150 countries is available from the Oxford University Research Archive and an overview is available as a Supplementary Data in the appendix 2. In the presentation of results, we focus on changes in the means of the costs of diet for regions grouped by income, in line with World Bank classification.

### Role of the funding source

The funders of the study had no role in study design, data collection, data analysis, data interpretation, or writing of the report.

## Results

**Figure 1:**
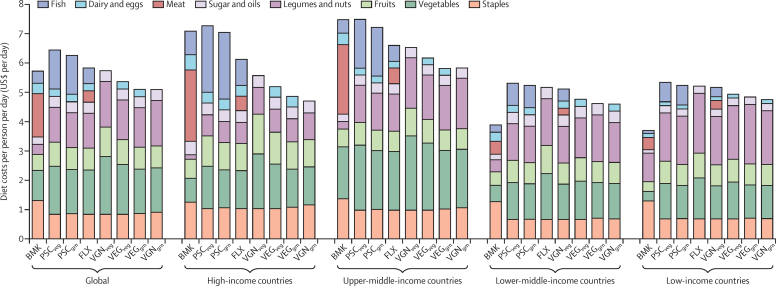
Costs of diets (US$ per day) in 2017 by dietary pattern, food group, and wrold regions grouped by income BMK=benchmark diets. FLX=flexitarian diets. PSC=pescatarian diets. VEG=vegetarian diets. VGN=vegan diets. Veg=diet variant high in fruits and vegetables. Grn=high-grain diet variant.

According to our estimates of 150 countries, the average cost of diets in 2017, including food wasted by households, was $5·7 (95% CI 3·9–7·6) per person per day, ranging from $3·7 (2·2–5·2) in low-income countries to $7·5 (5·3–9·7) in upper-middle-income countries (figure 1). In low-income and lower-middle-income countries, staple crops accounted for the greatest proportion of costs (33–35% across the two regions), followed by legumes and nuts (11–27%), meat (11% in each region), vegetables (9–14%), and fruits (9–12%). In comparison, in high-income and upper-middle-income countries, meat accounted for the greatest proportion of costs (32–34%), followed by staples (18% in both regions), vegetables (11–24%), and fruits (8–9%).

The alternative dietary patterns ranged from being 1–14% more expensive (high-veg pescatarian, high-grain pescatarian, flexitarian, and high-veg vegan) to 6–11% less expensive (vegetarian, high-grain vegetarian, high-grain and vegan) on average compared with current diets, with large variability across income regions (figure 1). In high-income and upper-middle-income countries, all dietary patterns, except for the high-veg pescatarian diets, were less expensive, with greatest cost reductions for the high-grain vegetarian and vegan diets (cost reductions of 22–34% across the two regions), followed by the high-veg vegetarian and vegan diets (17–27%), the flexitarian diets (12–14%), and the high-grain pescatarian diets (1–3% in each region). In lower-middle-income and low-income countries, all dietary patterns were more expensive (18–45%) in a similar order.

**Figure 2:**
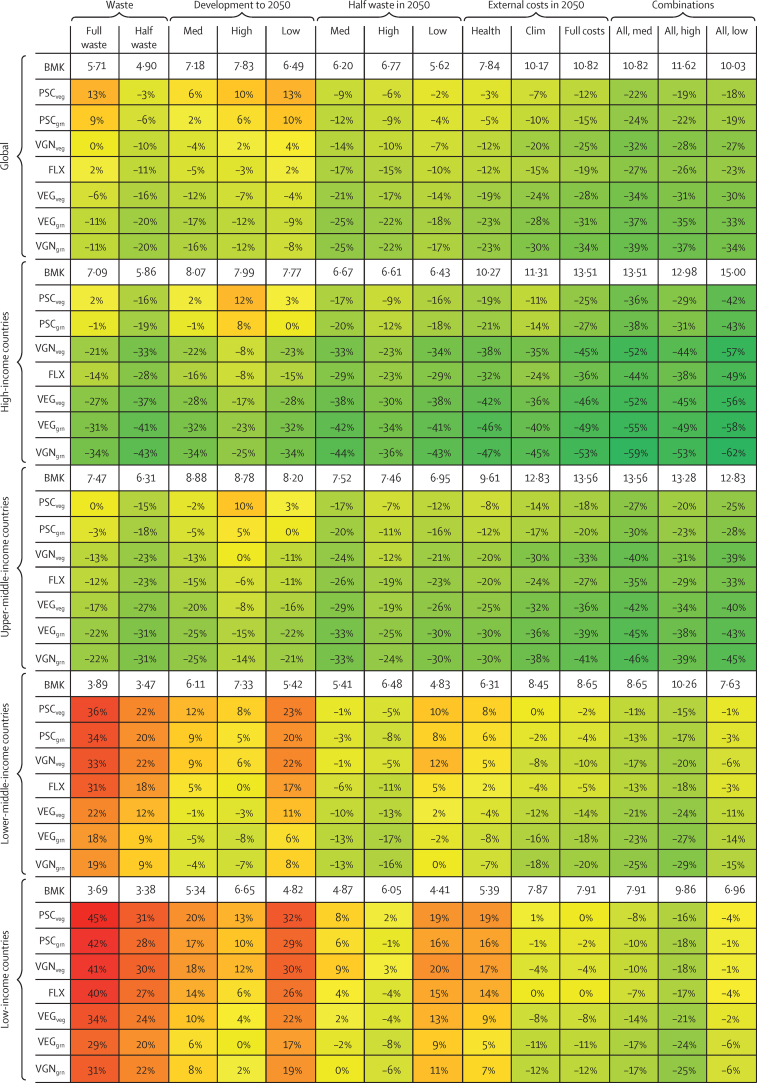
Cost of diets (US$ per day) for benchmark diets and relative percentage changes by dietary pattern, world region grouped by income, and food-system scenario The food-system scenarios consist of changes in food waste (full waste *vs* half waste), three socioeconomic development projections to 2050 (med *vs* high *vs* low), additional halving of food waste for these development projections (med *vs* high *vs* low under half waste in 2050), external cost estimates (health *vs* clim), and combinations of those scenarios for the three development projections (med *vs* high *vs* low). BMK=benchmark diets. FLX=flexitarian diets. PSC=pescatarian diets. VEG=vegetarian diets. VGN=vegan diets. Veg=diet variant high in fruits and vegetables. Grn=high-grain diet variant. Full waste=no change in waste. Half waste=halving of waste. Med=business-as-usual development (SSP2). High=more favourable development with greater income growth (SSP1). Low=less favourable development with lower income growth (SSP3). Health=health-care costs. Clim=climate-change costs.

Food waste at the household level accounted for 29% of the costs of current diets on average, ranging from 17% in low-income countries to 35% in high-income countries (appendix 1 p 36). Halving food waste, in line with the ambition expressed in the Sustainable Development Goals, reduced the costs of current diets by 14% on average (8–17% across income regions; figure 2). Combining dietary changes towards the different dietary patterns with reductions in food waste reduced the relative costs of those diets. Compared with current diets, this reduction in food waste for the alternative diets resulted in cost savings for all alternative dietary patterns in high-income and upper-middle-income countries (15–43% across diets), and on average across all income regions (3–20%), but the dietary patterns remained more expensive in lower-middle-income and low-income countries (9–31%).

As income increases, diets and food prices are projected to change, especially in low-income and lower-middle-income countries. Expenditure on staple crops is projected to decrease from 35% to 28% in low-income countries, and from 33% to 25% in lower-middle-income countries, in each case with concomitant increases in expenditure on meat and vegetables (appendix 1 p 37). Compared with these presumably more expensive diets in 2050, the high-grain vegetarian and vegan diets were lower in costs in lower-middle-income countries, and they came within a 6–8% difference in low-income countries in the business-as-usual projections and became comparable in costs for a more favourable socioeconomic development pathway (figure 2). Additionally, halving food waste in 2050 made several dietary patterns more affordable than benchmark diets in each region, in particular when combined with favourable socioeconomic development.

Accounting for the food-related costs of climate change and for the diet-related health-care costs increased the average costs of current diets by 12% and 4%, respectively, and the average costs of 2050 diets by 42% and 9%, respectively (figure 2). The combined increases were greater in high-income and upper-middle-income countries ($4·7–5·4 per day, 53–67% in 2050) than in low-income and lower-middle-income countries ($2·5–2·6 per day, 42–48% in 2050; appendix 1 pp 39–40). The different dietary patterns were both healthier and lower in greenhouse gas emissions than current diets (appendix 1 pp 38, 40), and the associated increases in costs were lower (on average 6–9% compared with 17% in 2017, and 18–28% compared with 51% in 2050), with lowest increases for the high-veg and high-grain vegan diets. Under this fuller cost accounting, the alternative dietary patterns were lower in costs than current diets on average (9–19% lower), except for pescatarian diets, and also lower in costs than 2050 diets, both on average (12–34% lower) and in each income region, except for high-veg pescatarian diets in low-income countries. Combining full-cost accounting with reductions in waste or with greater socioeconomic development resulted in further potential cost savings for all alternative dietary patterns in all income regions.

**Figure 3:**
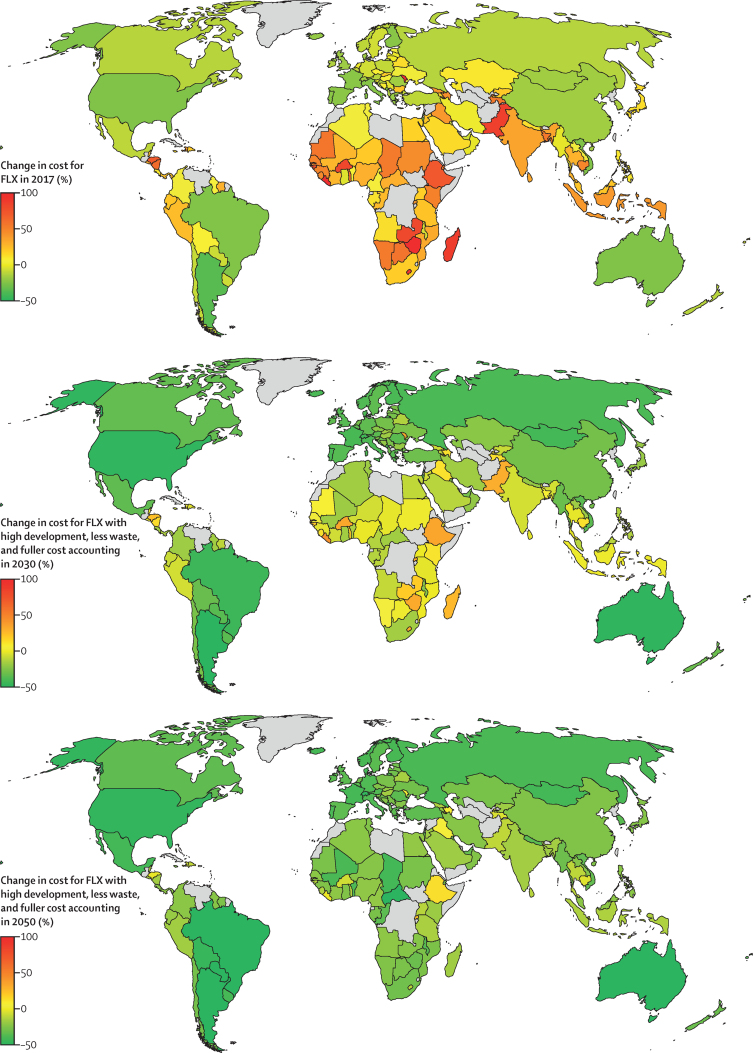
Change in cost for adoption of flexitarian diets in 2017, and in 2030 and 2050 when food-system measures are implemented Change in cost (%) include dietary changes to flexitarian diets in 2017 (top), and additinal food-system measures in 2030 (middle) and 2050 (bottom). The food-system measures consist of favourable socioeconomic development (SSP1), a halving of food waste, and a fuller cost accounting that includes health-change and climate-change costs in the price of foods. FLX=flexitarian diets.

At the country-level, the number of countries for which adoption of the different dietary patterns reduced the cost of diets (of the 150 included countries) ranged from 24 countries (16%, with a population of 2·6 billion people) for high-veg pescatarian diets to 81 countries (54%, 3·8–3·9 billion people) for high-grain vegan and vegetarian diets in 2017 (figure 3; appendix 1 p 42). This number increased to 97–101 countries (65–67%, 4·1 billion people) for high-grain vegan and vegetarian diets when food waste was halved, further to 116–118 countries (77–78%, 6·6 billion people) when combined with favourable socioeconomic development and assessed in 2030, and to 129 countries (86%, 6·9 billion people) when also combined with a fuller cost accounting. By 2050, the combination of those measures resulted in lower costs in almost all countries included in the analysis (up to 144–145 countries, 96–97%, 7·8 billion people).

## Discussion

We assessed the costs of dietary patterns that, in modelled studies, have been assessed as healthier and more sustainable than current diets, and were in line with the environmental limits of the food system.^2^, ^6^ Our analysis indicates that several of these dietary patterns are lower in cost than current diets in most high-income and many middle-income countries, but higher in cost in low-income countries. Reductions in food waste and future socioeconomic changes increased their relative affordability, especially in middle-income and some low-income countries, and a fuller cost accounting that included the diet-related costs of climate change and health care in the cost of diets further increased the relative affordability to most countries, including low-income countries.

Across the dietary patterns, the relative affordability was largest for vegetarian and vegan diets that focused on legumes and whole grains in place of animal products in current diets, and lowest for pescatarian diets that focused on fish and fruits and vegetables. Fish as a food group had one of the highest prices per calorie in the price data we used (appendix 1 p 35), which made pescatarian diets relatively costly. In comparison, grains and plant-based protein sources, such as legumes and nuts, had lower costs than vegetables and most animal products, which made the high-grain vegetarian and vegan diets relatively affordable. However, staple crops had one of the lowest costs of all foods, which made any deviation from current diets in low-income countries that are dominated by staple crops (and as a result lacking recommended quantities of many health-promoting foods) less affordable, if not complemented by reductions in food waste, socioeconomic changes, or a fuller accounting of the costs of diets.

Our analysis advances the current literature on the costs of healthy and sustainable diets. By using regionally comparable data on food prices and consumption, and a set of established healthy and sustainable dietary patterns, we were able to expand the scope of existing case studies and assess regional differences in a comparable and consistent manner. We also integrated a consistent analysis of the cost of different dietary patterns with an assessment of other changes in the food system, socioeconomic changes, and a fuller accounting of costs, which—to the best of our knowledge—has not been done previously.

But several caveats apply as well. First, our cost estimates can be considered conservative, despite our inclusion of household food waste. Our price data was representative of supermarket prices and did not account for the markups in restaurants, and our consumption data covered basic food categories which, while adequately capturing total consumption, did not allow us to explicitly account for the consumption and prices of highly processed food products and drinks. However, food consumption out of home, as well as highly processed and discretionary foods, often carry significant price premiums compared with their basic ingredients. Analysing the cost implications of changes in the consumption of ultra-processed foods, which in many countries constitute a major component of diets, is an important area of future research.

Second, our analysis of price feedbacks from dietary changes was limited to those associated with different socioeconomic trajectories. For example, along a business-as-usual pathway to 2050, global vegetable demand increased by 35% and prices by 29%, whereas vegetable demand increased by 85% and prices by 43% along a more favourable socioeconomic development pathway that included greater income growth and a greater degree of adoption of healthier diets (appendix 1 pp 25–26). However, if larger-scale changes to healthier and more sustainable diets were to occur more rapidly and simultaneously across the world, then the demand for nutritious plant-based foods, such as fruits and vegetables, could increase more than in the baseline trajectories. Greater changes in demand would then result in greater changes in prices in the absence of concomitant supply-side responses, such as support for agriculture research and progressive pricing support. Our results are, therefore, best interpreted from the perspective of individual regions or countries whose additional changes in demand are unlikely to change world-market prices beyond the range of existing socioeconomic projections.

Third, our valuation of the diet-related costs of climate change and health care is subject to high uncertainty and are best seen as indicative. In the analysis of health-care costs, we had to rely on a cost-transfer method that adjusted base costs that were measured for one region for changes in income and health expenditure to derive health-cost estimates for regions and years.^5^ In the analysis of climate-change costs, we aimed to reduce uncertainty in the social cost of carbon by using model estimates that were in line with stated policy objectives and using market-based discount rates.^36^ However, many uncertainties remain, including the feedback between changes in temperature and economic growth,^46^ and the rate of climate change adaptation and mitigation.^47^ In addition, there are many other impacts of the food system that are currently not reflected in food prices, including biodiversity impacts, as well as air and water pollution.^7^ The full cost of foods and diets is, therefore, probably considerably higher than our estimates that focused on two currently accounted cost components. However, also the cost savings for adopting healthier and more sustainable diets can be expected to be larger because those diets are often associated with joint reductions in multiple health and environmental impacts.^6^, ^7^, ^48^, ^49^

Compared with existing studies, our results generalise case-study results from several high-income countries according to which healthy or sustainable diets can be achieved without increases in cost.^10^, ^11^, ^12^, ^13^ While mirroring the global cost pattern of one specific dietary pattern that was identified in a global study published in 2020,^8^ our results also show the large spread in costs and the associated cost-saving potential across different healthy and sustainable dietary patterns. Our assessment of waste reduction, socioeconomic changes, and a fuller accounting of costs provide additional contextualisation, in particular for identifying measures that could improve the relative affordability of healthy and sustainable dietary patterns in low-income countries.

Our results are opposed to those of a meta-analysis of case studies from high-income countries that found that healthier diet patterns were more expensive than less healthy ones.^9^ In contrast to that study, we used a set of standardised diet scenarios that were not self-selected, we used regionally comparable food prices, and we focused on national averages instead of subnational case studies. Those aspects make our estimates indicative of the possible changes in the cost of diets for adoption of healthy and sustainable diets, whereas the findings of the meta-analysis are more indicative of the existing differences in food expenditure between those who consume healthier diets and those who consume unhealthier diets, something that might often be confounded by differences in retail choice. Our finding that many healthy and sustainable dietary patterns can reduce the cost of diets in high-income countries was robust under a wide variety of scenario assumptions, including year of analysis and dietary pattern.

Our findings have several policy implications. The findings for high-income and middle-income countries suggest that dietary change interventions that incentivise adoption of healthy and sustainable diets can help consumers in those countries reduce costs while, at the same time, contribute to fulfilling national climate change commitments and reduce public health spending. Some fiscal measures intended to incentivise dietary changes, such as health or environmentally motivated taxes,^50^, ^51^ have been portrayed as being potentially financially regressive for households. Our findings suggest that this does not need to be the case, and that progressive policy approaches can, when successful in changing diets, be financially progressive as well, and particularly so when they contribute to internalising some of the costs that are currently not accounted for. However, some additional considerations might be warranted to ensure the equitable use of tax revenues—eg, health promotion and support of low-income households can be considered,^7^ as well as integrating tax policies with other food policies, such as information campaigns and policies addressing food availability and accessibility.^52^

The findings for low-income countries suggest that any diversification of their current diets would increase costs, and that a long-term food-system perspective could help in identifying appropriate policy approaches, including a focus on waste reduction and development. However, while development policies could make diets more affordable, the relative increases in income are also expected to contribute to a transition to more western diets (with high proportions of animal products and ultra-processed foods) that are associated with increased burden of diet-related diseases and environmental pollution.^14^, ^53^ Our findings show that healthier and more sustainable dietary patterns are generally lower in costs than western diets (appendix 1 p 43), and they can be cost-competitive in low-income contexts in the long run if a health and environmentally sensitive policy environment and development strategies are in place. Supporting measures include consumer and food policies, agricultural incentives, and official development assistance, that take account of the health and environmental aspects of dietary change. Dietary changes to western diets instead of healthy and sustainable ones would impact greater costs on households, health-care systems, and the planet.

## Data sharing

The estimates of diet costs by country and region, commodity, and diet scenario will be deposited in the Oxford University Research Archive (at https://doi.org/10.5287/bodleian:bpPJMRzJo) and an overview is available as a Supplementary Data File in appendix 2.

## Declaration of interests

MS reports grants from Wellcome Trust (205212/Z/16/Z) and personal fees from the Global Panel on Agriculture and Food Systems for Nutrition, during the conduct of the study. MAC reports grants from Wellcome Trust (205212/Z/16/Z) during the conduct of the study. PW reports personal fees from the Global Panel on Agriculture and Food Systems for Nutrition, during the conduct of the study. MR reports grants from Wellcome Trust (205212/Z/16/Z), during the conduct of the study. PS reports grants from Wellcome Trust (205212/Z/16/Z) and the British Heart Foundation (FS/15/34/31656), during the conduct of the study. The authors had no other financial relationships with any organisations that might have an interest in the submitted work in the previous 3 years, and no other relationships or activities that could appear to have influenced the submitted work.
