# Correction to *Lancet Planet Health* 2021; 5: e797–807

**DOI:** 10.1016/S2542-5196(21)00316-8

**Published:** 2021-11-24

*Springmann M, Clark MA, Rayner M, Scarborough P, Webb P. The global and regional costs of healthy and sustainable dietary patterns: a modelling study.* Lancet Planet Health *2021;* 5: *e797–807*—The diet labels for lower middle-income and low-income countries in figure 1 were transposed during the production process, and Papua New Guinea has been added to the maps in figure 3. This correction has been made as of Nov 24, 2021.

