# Supplementary material for: The global and regional costs of healthy and sustainable dietary patterns: a modelling study
Source: Lancet Planet Health. 2021 Oct 27;5(11):e797–807. doi: 10.1016/S2542-5196(21)00251-5 (PMC8581186; doi:10.1016/S2542-5196(21)00251-5)
Supplement: Supplementary appendix 1 [file mmc1.pdf]

THE LANCET
Oncology

Supplementary appendix 1

This appendix formed part of the original submission and has been peer reviewed. We post it as supplied by the authors.

Supplement to: Springmann M, Clark MA, Rayner M, Scarborough P, Webb P. The global and regional costs of healthy and sustainable dietary patterns: a modelling study. *Lancet Oncol* 2021; published online Oct 26. http://dx.doi.org/10.1016/S2542-5196(21)00251-5.

The global and regional costs of healthy and sustainable dietary pattern: a modelling study

Appendix

Table of Contents

SI.1 Price data 2
SI.2 Consumption data 17
SI.3 Diet scenarios 20
SI.4 Projections of food demand and prices 24
SI.5 Health analysis and valuation 27
SI.6 Climate-change analysis and valuation 33
SI.7 Supplementary results 35
References 44

SI.1 Price data

For our study, we used country-specific price data of food commodities collected as part of the International Comparison Program (ICP). The ICP is a worldwide statistical initiative led by the World Bank under the auspices of the United Nations Statistical Commission, with the main objective of providing comparable price and volume measures of gross domestic product (GDP) and its expenditure aggregates among countries within and across regions. Through a partnership with international, regional, sub-regional and national agencies, the ICP collects and compares price data and GDP expenditures to estimate and publish purchasing power parities (PPPs) of the world’s economies. PPPs measure the total amount of goods and services that a single unit of a country’s currency can buy in another country. PPPs can thus be used to convert the cost of a basket of goods and service into a common currency while eliminating price level differences across countries. The latest available data is for the year 2017.

Economies participating in ICP collect prices for a selection of the goods and services, including food products. Prices are reported as national annual average prices in local currency, i.e. they represent the average of prices collected at regular intervals throughout the year by household consumption surveys. Each region develops its own list of regional items for the regional comparison, which includes region-specific items representative of the consumption pattern in the region, as well as items from a global core list. The latter has been developed for the specific purpose of linking the regional results into a global set of results by including products that can be priced across most regions. Structured product descriptions are used to define the price-determining characteristics of the products.

For our analysis, we used a total of 20,666 estimates of annual average prices in 179 countries, covering 463 food items including 144 items from the global-core list and 319 items from the regional lists. We aggregated the detailed list of food items into a list of 31 food groups which we used to construct the diet scenarios. In line with the definition of the diet scenarios, we focused on those categories that are related to foods and can be expressed in primary commodity equivalents (i.e., as basic ingredients). SI Table 1 lists the regional coverage of the ICP data, and SI Table 2 lists the aggregation of food commodities into the food groups used for the analysis.

For the aggregation, we paired each item with its caloric content (to control for difference in processing and edible fractions), and converted the prices from local currency to USD. For the calorie conversion, we used data from the FoodData Central database maintained by the US Department of Agriculture to pair each food item with its caloric content. The price conversion was based on PPPs at the basic heading level which, for the food items used in the analysis, describes 24 groups of similar well-defined goods or services at the lowest level (i.e., most detailed) aggregation for which PPPs are calculated.

SI Table 1. Regional and country-level coverage of ICP data

Code	Name	Regional Program
ALB	Albania	Eurostat-OECD
DZA	Algeria	ICP Africa
AGO	Angola	ICP Africa
AIA	Anguilla	ICP Latin America and the Caribbean
ATG	Antigua and Barbuda	ICP Latin America and the Caribbean
ARG	Argentina	ICP Latin America and the Caribbean
ARM	Armenia	ICP Commonwealth of Independent States
ABW	Aruba	ICP Latin America and the Caribbean
AUS	Australia	Eurostat-OECD
AUT	Austria	Eurostat-OECD
AZE	Azerbaijan	ICP Commonwealth of Independent States
BHS	Bahamas, The	ICP Latin America and the Caribbean
BHR	Bahrain	ICP Western Asia
BGD	Bangladesh	ICP Asia and the Pacific
BRB	Barbados	ICP Latin America and the Caribbean
BLR	Belarus	ICP Commonwealth of Independent States
BEL	Belgium	Eurostat-OECD
BLZ	Belize	ICP Latin America and the Caribbean
BEN	Benin	ICP Africa
BMU	Bermuda	ICP Latin America and the Caribbean
BTN	Bhutan	ICP Asia and the Pacific
BOL	Bolivia	ICP Latin America and the Caribbean
BON	Bonaire	ICP Latin America and the Caribbean
BIH	Bosnia and Herzegovina	Eurostat-OECD
BWA	Botswana	ICP Africa
BRA	Brazil	ICP Latin America and the Caribbean
BRN	Brunei Darussalam	ICP Asia and the Pacific
BGR	Bulgaria	Eurostat-OECD
BFA	Burkina Faso	ICP Africa
BDI	Burundi	ICP Africa
KHM	Cambodia	ICP Asia and the Pacific
CMR	Cameroon	ICP Africa
CAN	Canada	Eurostat-OECD

CPV	Cabo Verde	ICP Africa
CYM	Cayman Islands	ICP Latin America and the Caribbean
CAF	Central African Republic	ICP Africa
TCD	Chad	ICP Africa
CHL	Chile	Eurostat-OECD
CHN	China	ICP Asia and the Pacific
COL	Colombia	Eurostat-OECD
COM	Comoros	ICP Africa
COD	Congo, Dem. Rep.	ICP Africa
COG	Congo, Rep.	ICP Africa
CRI	Costa Rica	Eurostat-OECD
CIV	Côte d'Ivoire	ICP Africa
HRV	Croatia	Eurostat-OECD
CUW	Curaçao	ICP Latin America and the Caribbean
CYP	Cyprus	Eurostat-OECD
CZE	Czech Republic	Eurostat-OECD
DNK	Denmark	Eurostat-OECD
DJI	Djibouti	ICP Africa
DMA	Dominica	ICP Latin America and the Caribbean
DOM	Dominican Republic	ICP Latin America and the Caribbean
ECU	Ecuador	ICP Latin America and the Caribbean
EGY	Egypt, Arab Rep. (AFR)	ICP Africa & ICP Western Asia (dual participation)
EGZ	Egypt, Arab Rep. (WAS)	ICP Africa & ICP Western Asia (dual participation)
SLV	El Salvador	ICP Latin America and the Caribbean
GNQ	Equatorial Guinea	ICP Africa
EST	Estonia	Eurostat-OECD
SWZ	Eswatini	ICP Africa
ETH	Ethiopia	ICP Africa
FJI	Fiji	ICP Asia and the Pacific
FIN	Finland	Eurostat-OECD
FRA	France	Eurostat-OECD
GAB	Gabon	ICP Africa
GMB	Gambia, The	ICP Africa
GEO	Georgia	ICP Special Participation
DEU	Germany	Eurostat-OECD
GHA	Ghana	ICP Africa
GRC	Greece	Eurostat-OECD
GRD	Grenada	ICP Latin America and the Caribbean
GIN	Guinea	ICP Africa
GNB	Guinea-Bissau	ICP Africa
GUY	Guyana	ICP Latin America and the Caribbean
HTI	Haiti	ICP Latin America and the Caribbean
HND	Honduras	ICP Latin America and the Caribbean
HKG	Hong Kong SAR, China	ICP Asia and the Pacific
HUN	Hungary	Eurostat-OECD
ISL	Iceland	Eurostat-OECD
IND	India	ICP Asia and the Pacific
IDN	Indonesia	ICP Asia and the Pacific

IRN	Iran, Islamic Rep.	ICP Special Participation
IRQ	Iraq	ICP Western Asia
IRL	Ireland	Eurostat-OECD
ISR	Israel	Eurostat-OECD
ITA	Italy	Eurostat-OECD
JAM	Jamaica	ICP Latin America and the Caribbean
JPN	Japan	Eurostat-OECD
JOR	Jordan	ICP Western Asia
KAZ	Kazakhstan	ICP Commonwealth of Independent States
KEN	Kenya	ICP Africa
KOR	Korea, Rep.	Eurostat-OECD
KWT	Kuwait	ICP Western Asia
KGZ	Kyrgyz Republic	ICP Commonwealth of Independent States
LAO	Lao PDR	ICP Asia and the Pacific
LVA	Latvia	Eurostat-OECD
LSO	Lesotho	ICP Africa
LBR	Liberia	ICP Africa
LTU	Lithuania	Eurostat-OECD
LUX	Luxembourg	Eurostat-OECD
MDG	Madagascar	ICP Africa
MWI	Malawi	ICP Africa
MYS	Malaysia	ICP Asia and the Pacific
MDV	Maldives	ICP Asia and the Pacific
MLI	Mali	ICP Africa
MLT	Malta	Eurostat-OECD
MRT	Mauritania	ICP Africa
MUS	Mauritius	ICP Africa
MEX	Mexico	Eurostat-OECD
MDA	Moldova	ICP Commonwealth of Independent States
MNG	Mongolia	ICP Asia and the Pacific
MNE	Montenegro	Eurostat-OECD
MSR	Montserrat	ICP Latin America and the Caribbean
MAR	Morocco (AFR)	ICP Africa & ICP Western Asia (dual participation)
MAS	Morocco (WAS)	ICP Africa & ICP Western Asia (dual participation)
MOZ	Mozambique	ICP Africa
MMR	Myanmar	ICP Asia and the Pacific
NAM	Namibia	ICP Africa
NPL	Nepal	ICP Asia and the Pacific
NLD	Netherlands	Eurostat-OECD
NZL	New Zealand	Eurostat-OECD
NIC	Nicaragua	ICP Latin America and the Caribbean
NER	Niger	ICP Africa
NGA	Nigeria	ICP Africa
MKD	North Macedonia	Eurostat-OECD
NOR	Norway	Eurostat-OECD
OMN	Oman	ICP Western Asia
PAK	Pakistan	ICP Asia and the Pacific
PAN	Panama	ICP Latin America and the Caribbean

PRY	Paraguay	ICP Latin America and the Caribbean
PER	Peru	ICP Latin America and the Caribbean
PHL	Philippines	ICP Asia and the Pacific
POL	Poland	Eurostat-OECD
PRT	Portugal	Eurostat-OECD
QAT	Qatar	ICP Western Asia
ROU	Romania	Eurostat-OECD
RUS	Russian Federation	Eurostat-OECD & ICP Commonwealth of Independent States (dual participation)
RWA	Rwanda	ICP Africa
STP	São Tomé and Principe	ICP Africa
SAU	Saudi Arabia	ICP Western Asia
SEN	Senegal	ICP Africa
SRB	Serbia	Eurostat-OECD
SYC	Seychelles	ICP Africa
SLE	Sierra Leone	ICP Africa
SGP	Singapore	ICP Asia and the Pacific
SXM	Sint Maarten (Dutch part)	ICP Latin America and the Caribbean
SVK	Slovak Republic	Eurostat-OECD
SVN	Slovenia	Eurostat-OECD
ZAF	South Africa	ICP Africa
ESP	Spain	Eurostat-OECD
LKA	Sri Lanka	ICP Asia and the Pacific
KNA	St. Kitts and Nevis	ICP Latin America and the Caribbean
LCA	St. Lucia	ICP Latin America and the Caribbean
VCT	St. Vincent and the Grenadines	ICP Latin America and the Caribbean
SDN	Sudan (AFR)	ICP Africa & ICP Western Asia (dual participation)
SDO	Sudan (WAS)	ICP Africa & ICP Western Asia (dual participation)
SUR	Suriname	ICP Latin America and the Caribbean
SWE	Sweden	Eurostat-OECD
CHE	Switzerland	Eurostat-OECD
TWN	Taiwan, China	ICP Asia and the Pacific
TJK	Tajikistan	ICP Commonwealth of Independent States
TZA	Tanzania	ICP Africa
THA	Thailand	ICP Asia and the Pacific
TGO	Togo	ICP Africa
TTO	Trinidad and Tobago	ICP Latin America and the Caribbean
TUN	Tunisia	ICP Africa
TUR	Turkey	Eurostat-OECD
TCA	Turks and Caicos Islands	ICP Latin America and the Caribbean
UGA	Uganda	ICP Africa
UKR	Ukraine	ICP Special Participation
ARE	United Arab Emirates	ICP Western Asia
GBR	United Kingdom	Eurostat-OECD
USA	United States	Eurostat-OECD
URY	Uruguay	ICP Latin America and the Caribbean
VNM	Vietnam	ICP Asia and the Pacific
VGB	British Virgin Islands	ICP Latin America and the Caribbean

PSE	West Bank and Gaza	ICP Western Asia
ZMB	Zambia	ICP Africa
ZWE	Zimbabwe	ICP Africa

SI Table 2. Aggregation from food items to the food groups used in the analysis from the ICP global-core list (GLB) and the regional lists of ICP regional program's in the Commonwealth of Independent States (CIS), Africa (AFR), Western Asia (WAS), Asia and the Pacific (ASI), and Latin America and the Caribbean (LAC).

Item name	Food group	Coverage
Long-grain rice, parboiled, WKB	rice	GLB
Long-grain rice, not parboiled, WKB	rice	GLB
Long grain rice, family pack, WKB	rice	GLB
Jasmine rice, WKB	rice	GLB
Basmati rice, WKB	rice	GLB
Broken rice, 25%, BNR	rice	GLB
Medium-grain rice, BNR	rice	GLB
Brown rice, family pack, BL	rice	GLB
Short-grain rice, BNR	rice	GLB
Sticky rice, WKB	rice	GLB
Long-grain rice, not parboiled, BL	rice	CIS
Long-grain rice, parboiled, in cooking bags, WKB	rice	CIS
Round-grain Rice, WKB	rice	CIS
Risotto Rice, WKB	rice	CIS
Long-grained rice-25 to 50 KG,BNR	rice	AFR
Egyptian rice	rice	WAS
Sun White Rice - Australia	rice	WAS
Uncle Ben's rice	rice	WAS
Brown rice, loose	rice	ASI
White rice #1, BNR	rice	ASI
White rice #3, BNR	rice	ASI
White rice #5, BNR	rice	ASI
White rice #7, Prepacked, BL	rice	ASI
White rice #8, Prepacked, BL	rice	ASI
White rice #9, Prepacked, BL	rice	ASI
White rice #10, Prepacked, BL	rice	ASI
Premium rice #1, Prepacked, BL	rice	ASI
Premium rice #2, Prepacked, BL	rice	ASI
Premium rice #3, BNR	rice	ASI
Premium rice #4, BNR	rice	ASI
Rice flour, Atta, WKB	rice	ASI
Wheat flour, not self-rising, BL	wheat	GLB
Wheat semolina (suji), WKB	wheat	GLB
Baguette, BNR	wheat	GLB
Bread, white, loaf, BNR	wheat	GLB

Bread, whole wheat, loaf, BNR	wheat	GLB
Roll, BNR	wheat	GLB
Bread, white, sliced, WKB	wheat	GLB
Pita bread, BNR	wheat	GLB
Short pasta, BL	wheat	GLB
Spaghetti, WKB	wheat	GLB
Dried noodles, WKB	wheat	GLB
Instant noodles, any flavor, WKB	wheat	GLB
Vermicelli, BL	wheat	GLB
Short pasta, WKB	wheat	GLB
Couscous, BNR	wheat	GLB
Spaghetti, BARILLA	wheat	GLB
Wheat flour, WKB	wheat	CIS
Roll, multicorn	wheat	CIS
Bread, white,Large Loaf	wheat	CIS
Bread, white, industrially packed, WKB	wheat	CIS
Bread, white, toast,Large pack, WKB	wheat	CIS
Bread, mixed	wheat	CIS
Bread, multicorn	wheat	CIS
Bread, multicorn, industrially packed, WKB	wheat	CIS
National bread (johnny-cake) (CIS)	wheat	CIS
Spaghetti, BL	wheat	CIS
Pasta, with eggs, WKB	wheat	CIS
Bread unpacked	wheat	LAC
Round bread	wheat	AFR
Sliced brown bread (AFR)	wheat	AFR
Sweet Bread	wheat	AFR
Sliced brown bread (WAS)	wheat	WAS
Burger Bread	wheat	WAS
Samoon Bread	wheat	WAS
Lebanese Bread	wheat	WAS
Brown Flour	wheat	WAS
Wheat flour, loose, BNR	wheat	ASI
Wholemeal flour, Atta, BL	wheat	ASI
Bread, white, unsliced, WKB	wheat	ASI
Roll or bun, Prepacked, BNR	wheat	ASI
Lasagne (sheets)	wheat	AFR
Egg noodles, WKB	wheat	ASI
Fresh rice noodles, BL	wheat	ASI
Corn (maize) flour, white, WKB	maize	GLB
Maize Flour, Yellow	maize	AFR
Maize Flour White (Maizena)	maize	AFR
Maize grains, White	maize	AFR
Corn	maize	WAS
STARCH	maize	WAS
Corn (maize) flour, loose, BL	maize	ASI
Maize, BL	maize	ASI
Oats, rolled, WKB	othr_grains	GLB

Buckwheat (CIS)	othr_grains	CIS
Millet (CIS)	othr_grains	CIS
Hard Loose Bulgur	othr_grains	WAS
Millet, Sorghum, BL	othr_grains	ASI
Oats, Quaker	othr_grains	ASI
Beef, centerBrisket, withBones (B2)	beef	CIS
Beef, Silverside (F2a)	beef	CIS
Beef, Sirloin steak (H1)	beef	CIS
Veal, schnitzel/escalope (A5)	beef	CIS
Veal, loin (B2)	beef	CIS
Beef, fillet, tenderloin	beef	GLB
Beef, rump steak	beef	GLB
Beef, center brisket	beef	GLB
Beef, cubes for stew or curry	beef	GLB
Beef, with bones	beef	GLB
Beef, minced	beef	GLB
Veal chops	beef	GLB
Veal breast, with bones	beef	GLB
Beef without bones	beef	AFR
Burger	beef	WAS
Round steak	beef	ASI
Sirloin steak	beef	ASI
Buffalo, without bones, non-specific cut	beef	ASI
Beef, without bones, non-specific cut	beef	ASI
Veal, with bones	beef	ASI
Beef, fillet, frozen, tenderloin	beef	ASI
Pork, schnitzel/escalope (A)	pork	CIS
Pork, belly (C)	pork	CIS
Pork, collar (B1)	pork	CIS
Pork, joint piece for roasting (B2)	pork	CIS
Pork, loin chop	pork	GLB
Pork, fillet	pork	GLB
Pork, shoulder	pork	GLB
Pork, ribs	pork	GLB
Pork thigh, with bones	pork	ASI
Pork loin, without bones	pork	ASI
Pork, without bones, non-specific cut	pork	ASI
Pork, with bones, non-specific cut	pork	ASI
Lamb, hindLeg (hindquarters A)	lamb	CIS
Lamb, whole leg	lamb	GLB
Lamb, chops	lamb	GLB
Mutton, mixed cut	lamb	GLB
Goat, mixed cut, with bones	lamb	GLB
Mutton chop	lamb	AFR
Live Sheep	lamb	AFR
Live Goat	lamb	AFR
Goat leg	lamb	ASI
Mutton chops	lamb	ASI

Chicken, for roasting, free range	poultry	CIS
Turkey breast, fillet	poultry	CIS
Chicken, whole, fresh	poultry	GLB
Chicken, whole, frozen	poultry	GLB
Chicken breast, without skin and bones	poultry	GLB
Chicken legs	poultry	GLB
Chicken, live	poultry	GLB
Chicken breast, with skin and bones	poultry	GLB
Chicken, Traditionally bred, live	poultry	AFR
Chicken wings (WAS)	poultry	WAS
Chicken burger	poultry	WAS
Native house chicken, fresh	poultry	ASI
Whole duck, fresh	poultry	ASI
Chicken wings (ASI)	poultry	ASI
Chicken, non-specific cuts, fresh	poultry	ASI
Chicken, non-specific cuts, frozen	poultry	ASI
Sausage, Frankfurter/Wiener type, artificial skin, WKB	pork	CIS
Sausage, fresh and raw, sold loose	pork	CIS
Cooked pork sausage, country typical variety, sold loose	pork	CIS
Ham, air dried, sold loose	pork	CIS
Ham, from the thigh, cooked and smoked, sold loose	pork	CIS
Ham, turkey, WKB	pork	CIS
Salami, sold loose	pork	CIS
Salami, WKB	pork	CIS
Grilled/roasted chicken	poultry	CIS
Chicken nuggets/dippers, frozen, WKB	poultry	CIS
Pork ham, pressed, WKB	pork	GLB
Bacon, smoked, WKB	pork	GLB
Beef liver, BNR	beef	GLB
Corned beef, WKB	beef	GLB
Canned chicken	poultry	AFR
Sausages, Whole/Frankfurter	pork	CAR
Mutton Tripes	lamb	AFR
Sausage	pork	AFR
Beef Merguez (spiced)	beef	AFR
Beef ham	beef	AFR
Lamb Liver	lamb	WAS
Luncheon Chicken	poultry	WAS
Luncheon Meat	pork	WAS
BEEF SAUSAGE	beef	WAS
Camel Meat	lamb	WAS
Turkey	poultry	WAS
Pork liver, BNR	pork	ASI
Mutton/goat liver, BNR	lamb	ASI
Breakfast sausage, chicken, BNR	poultry	ASI
Sliced ham, pork, WKB	pork	ASI
Canned beef, chunks, WKB	beef	ASI
Rainbow-Trout (Salmo gairdneri)	fish_freshw	CIS

Cod (Gadus morhua), frozen, WKB	fish_demrs	CIS
Hake (Merluccius merluccius), Alaska Pollock (Theragra chalcogr	fish_demrs	CIS
Pangasius catfish (Pangasius hypophthalmus), fillet, frozen, BL	fish_freshw	CIS
Calamari rings, frozen, WKB	shellfish	CIS
Carp	fish_freshw	GLB
Mackerel, un-cleaned	fish_pelag	GLB
Sea bass	fish_other	GLB
Tuna steaks	fish_pelag	GLB
Shrimps, whole, fresh	shellfish	GLB
Shrimps, peeled, frozen	shellfish	GLB
Squid	shellfish	GLB
Cod (gadus morhua)	fish_demrs	GLB
Red snapper	fish_other	GLB
Sea crab	shellfish	GLB
Tilapia	fish_freshw	GLB
Black pomfret	fish_pelag	GLB
Mullet	fish_demrs	GLB
Fresh Small Sardines	fish_pelag	AFR
Tuna fish fresh	fish_pelag	WAS
Grouper (Hamour) fish	fish_other	WAS
Capitaine	fish_other	AFR
Red Snapper (AFR)	fish_other	AFR
Giant Shrimp	shellfish	WAS
Safi	fish_other	WAS
Lobster	shellfish	WAS
Catfish	fish_freshw	ASI
Spanish Mackerel	fish_pelag	ASI
Sole	fish_other	ASI
Tuna steak	fish_pelag	ASI
White pomfret	fish_other	ASI
Mud crab	shellfish	ASI
Sea lobster	shellfish	ASI
Prawn/Shrimp, small	shellfish	ASI
Prawn/Shrimp, medium	shellfish	ASI
Squid, small	shellfish	ASI
Small fresh fish	fish_pelag	ASI
Tuna	fish_pelag	ASI
Smoked mackerel (Scomber scombrus), fillet, WKB	fish_pelag	CIS
Tinned sardines, in olive oil, with skin and bones, WKB	fish_other	CIS
Tinned pink tuna (Skipjack, Thunnus Thynn, Albacares = yellow f	fish_pelag	CIS
Tinned tuna flakes, in vegetable oil, BL	fish_pelag	CIS
Breaded fish fillet (Pollock), 2 - 4 pieces, frozen, WKB	fish_demrs	CIS
Breaded fish fillet (Cod), 2 - 5 pieces, frozen, WKB	fish_demrs	CIS
Fish fingers, from fillet, WKB	fish_demrs	CIS
Fish fingers, BL	fish_demrs	CIS
Salty herring (CIS)	fish_pelag	CIS
Salmon in natural juice, WKB (CIS)	fish_freshw	CIS
Canned sprats in oil, WKB (CIS)	fish_pelag	CIS

Sardines, tinned, with skin, in vegetable oil, WKB	fish_pelag	GLB
Tuna flakes, tinned, WKB	fish_pelag	GLB
Mackerel fillet, tinned, in vegetable oil, WKB	fish_pelag	GLB
Mackerel fillet, tinned, in tomato sauce, WKB	fish_pelag	CAR
Cold-smoked salmon, WKB	fish_pelag	GLB
Dried shrimps, BNR	shellfish	GLB
Dried sardines, BNR	fish_pelag	AFR
Dried small fish, BNR	fish_pelag	AFR
Dried Machoiron,BNR	fish_freshw	AFR
Smoked shrimps/prawns, BNR	shellfish	AFR
Tuna in vegetable oil, exclude Tuna Steaks, WKB	fish_pelag	AFR
Sardines in tomato sauce,WKB	fish_pelag	AFR
Mackerel in vegetable oil, WKB	fish_pelag	AFR
Fissikh	fish_demrs	WAS
Salted and semi-dried fish, BL	fish_pelag	ASI
Fishball, BNR	fish_pelag	ASI
Milk, fresh, unskimmed, BNR (CIS)	milk	CIS
Milk, UHT, semi-skimmed, WKB	milk	CIS
Milk, UHT, semi-skimmed, BL	milk	CIS
Fresh cream (CIS)	cream	CIS
Milk, fresh, unskimmed, WKB	milk	GLB
Milk, UHT, unskimmed, WKB	milk	GLB
Milk, fresh, low-fat, WKB	milk	GLB
Buffalo milk, not pasteurized, BL	milk	ASI
Sour cream / crème epaisse / smetana, WKB	cream	CIS
Cream for whipping, WKB	cream	CIS
Milk, powdered, WKB	milk	GLB
Sour cream, WKB	cream	GLB
Powdered milk, WKB	milk	ASI
Cheese, processed, spreadable, industrially packed, WKB	cheese	CIS
Cheese, blue, industrially packed, WKB	cheese	CIS
Cheese, Edam or Gouda type, young, industrially packed, BL	cheese	CIS
Cheese, Emmental type, industrially packed, WKB	cheese	CIS
Brynza (White cheese) (CIS)	cheese	CIS
Cheese, Feta type, industrially packed, WKB	cheese	CIS
Cheese, Gouda type, industrially packed, WKB	cheese	CIS
Cheese, Mozzarella, industrially packed, WKB	cheese	CIS
Cheese, Parmesan type, dehydrated & grated, industrially packed	cheese	CIS
Cheese, cheddar, WKB	cheese	GLB
Cream cheese, WKB	cheese	GLB
Cheese, processed, WKB	cheese	GLB
Cheese, camembert type, WKB	cheese	GLB
Cheese, gouda type, WKB	cheese	GLB
Tofu, WKB	legumes	GLB
Fresh cheese emmental, BL	cheese	AFR
Fresh cheese edam, BL	cheese	AFR
white cheese	cheese	WAS
Labneh	cheese	WAS

Cheese spread, WKB	cheese	ASI
Local cheese, WKB	cheese	ASI
Chicken eggs, free range	eggs	CIS
Chicken eggs, caged hen, large size	eggs	GLB
Chicken eggs, caged hen, medium size	eggs	GLB
Eggs, traditional production	eggs	AFR
Chicken egg, caged hen, large size	eggs	ASI
Chicken eggs, caged hen, large size, loose	eggs	ASI
Salted duck egg	eggs	ASI
Butter, unsalted,BL	butter	CIS
Butter, unsalted, WKB	butter	GLB
Butter, salted, WKB	butter	GLB
Ghee / clarified butter, WKB	butter	GLB
Butter, sold loose	butter	AFR
Ghee	butter	WAS
Ghee, cow/buffalo, WKB	butter	ASI
Olive oil, extra virgin, BL	oil_veg	CIS
Sunflower oil, WKB	oil_veg	CIS
Cottonseed oil (CIS)	oil_veg	CIS
Sunflower oil, BL	oil_veg	GLB
Olive oil, extra virgin, WKB	oil_veg	GLB
Palm oil, WKB	oil_palm	GLB
Soybean oil, WKB	oil_veg	GLB
Peanut oil, WKB	oil_veg	GLB
Vegetable oil, WKB	oil_veg	GLB
Corn oil, WKB	oil_veg	GLB
Palm oil unrefined, BL	oil_palm	AFR
Tahina	treenuts	WAS
Corn Oil	oil_veg	WAS
Peanut butter, WKB	groundnuts	ASI
Olive oil, standard, WKB	oil_veg	ASI
Coconut oil, BL	oil_veg	ASI
Fresh apples, Golden Delicious or Granny Smith	fruits_temp	CIS
Fresh mandarines	fruits_trop	CIS
Fresh pears (CIS)	fruits_temp	CIS
Fresh kiwis	fruits_temp	CIS
Fresh strawberries (CIS)	fruits_temp	CIS
Fresh apple, red delicious	fruits_temp	GLB
Fresh bananas, standard	fruits_starch	GLB
Fresh grapes, white	fruits_temp	GLB
Fresh grapefruit	fruits_trop	GLB
Fresh oranges	fruits_trop	GLB
Fresh papaya	fruits_trop	GLB
Fresh pineapples	fruits_trop	GLB
Fresh lemons	fruits_trop	GLB
Fresh mangoes	fruits_trop	GLB
Fresh watermelon	fruits_trop	GLB
Plantains, Fresh Ripe	fruits_starch	CAR

Fresh apples, typical local variety	fruits_temp	GLB
Plantains, Fresh Green	fruits_starch	CAR
Fresh peaches	fruits_temp	GLB
Bananas, Fresh Green	fruits_starch	CAR
Fresh melon	fruits_trop	GLB
Large Mango (Grafted)	fruits_trop	AFR
Banana, short finger length	fruits_starch	AFR
Coconut	fruits_trop	AFR
Fresh Plum (Al-Barkouk)	fruits_temp	WAS
Fresh Pears (WAS)	fruits_temp	WAS
Fresh Clementine (Yussufi Orange)	fruits_trop	WAS
Fresh Cherries	fruits_temp	WAS
Fresh Apricots	fruits_temp	WAS
Fresh Strawberries (WAS)	fruits_temp	WAS
Fresh Dates	fruits_temp	WAS
Fresh Pomegranate	fruits_trop	WAS
Fresh Figs	fruits_trop	WAS
Fresh Guava	fruits_trop	WAS
Lime	fruits_trop	ASI
Grapes, violet, with seed	fruits_trop	ASI
Coconut, young green	fruits_trop	ASI
Tamarind	fruits_trop	ASI
Frozen strawberries, WKB	fruits_temp	CIS
Frozen raspberries, WKB	fruits_temp	CIS
Frozen berries, mixed, BL	fruits_temp	CIS
Dried almonds, peeled, WKB	treenuts	CIS
Dried walnuts, WKB	treenuts	CIS
Raisins, WKB	fruits_temp	CIS
Raisins, BL	fruits_temp	CIS
Dried apricots (CIS)	fruits_temp	CIS
Tinned peaches, in syrup, WKB	fruits_temp	CIS
Tinned pears, in syrup, WKB	fruits_temp	CIS
Fruit cocktail, in syrup, WKB	fruits_trop	CIS
Walnuts, inshell (CIS)	treenuts	CIS
Tinned pineapple, whole slices, BL	fruits_trop	GLB
Dried almonds, BL	treenuts	GLB
Roasted groundnuts (peanuts), WKB	groundnuts	GLB
Mixed fruits, in syrup, WKB	fruits_temp	GLB
Dried dates, WKB	fruits_temp	GLB
Natural Groundnuts, BL	groundnuts	AFR
Walnuts	treenuts	WAS
Cashewnuts	treenuts	WAS
Raisin	fruits_temp	WAS
Tamarind, preserved, BL	fruits_trop	ASI
Fresh broccoli	vegetables	CIS
Fresh cabbage lettuce, round, soft leaves	vegetables	CIS
Fresh courgettes	vegetables	CIS
Fresh cultivated mushrooms, white, whole	vegetables	CIS

Fresh leek	vegetables	CIS
Fresh tomato cluster	vegetables	CIS
Fresh cucumber	vegetables	GLB
Fresh green pepper	vegetables	GLB
Fresh carrots	vegetables	GLB
Fresh onions	vegetables	GLB
Fresh maize (corn) ears	vegetables	GLB
Fresh tomatoes, round	vegetables	GLB
Fresh cabbage, green	vegetables	GLB
Fresh iceberg lettuce	vegetables	GLB
Fresh avocado	vegetables	GLB
Fresh eggplants (aubergines)	vegetables	GLB
Fresh cauliflower	vegetables	GLB
Fresh spinach	vegetables	GLB
Fresh chilies, long	vegetables	GLB
Pumpkin (CAR)	vegetables	CAR
Ginger, mature	vegetables	GLB
Garlic, white	vegetables	GLB
Green Beans	legumes	AFR
Spotted beans	legumes	AFR
Peas (AFR)	legumes	AFR
Green beans (Pulses)	legumes	WAS
Okra	vegetables	WAS
Peas (WAS)	legumes	WAS
Dhal, Khesari, BL	legumes	ASI
Dhal, Musur, BL	legumes	ASI
Dhal, Split Peas, BL	legumes	ASI
Round red raddish	vegetables	WAS
Zucchini	vegetables	WAS
Mloukhia	vegetables	WAS
Lettuce	vegetables	WAS
Mint leaves	vegetables	WAS
Spinach Chinese	vegetables	ASI
Water spinach	vegetables	ASI
Pumpkin (ASI)	vegetables	ASI
Radish, white	vegetables	ASI
Fresh potatoes, industrially packed	roots	CIS
Fresh potatoes, brown	roots	GLB
Fresh sweet potatoes	roots	GLB
Fresh cassava / manioc / yuca	roots	GLB
Fresh potatoes, white	roots	ASI
Fresh taro	roots	ASI
Frozen green beans, fine, WKB	legumes	CIS
Frozen mixed vegetables, natural, WKB	vegetables	CIS
Frozen mixed vegetables, natural, BL	vegetables	CIS
Frozen peas, small/fine, WKB	legumes	CIS
Frozen spinach, natural, WKB	vegetables	CIS
Tomato puree (Passata di Pomodoro), WKB	vegetables	CIS

Chopped tomatoes, WKB	vegetables	CIS
Chopped tomatoes, BL	vegetables	CIS
Mushrooms, slices in salt water, BL	vegetables	CIS
Green olives, without stones, WKB	vegetables	CIS
Pickled gherkins, WKB	vegetables	CIS
Dried peas, WKB (CIS)	legumes	CIS
White beans, dried, BL	legumes	GLB
White beans in tomato sauce, tinned, HEINZ	legumes	GLB
Green olives, with stones, WKB	vegetables	GLB
Potato chips, WKB	roots	GLB
French fries, frozen, WKB	roots	GLB
Tomato paste, WKB	vegetables	GLB
Green peas, tinned, WKB	legumes	GLB
Sweet corn (maize), tinned, BL	vegetables	GLB
Canned black beans	legumes	LAC
Lentils, dried, WKB	legumes	GLB
Red Kidney beans, dried	legumes	CAR
Button mushrooms, tinned, WKB	vegetables	GLB
Tomato paste (Large), WKB	vegetables	AFR
Peppers, paste, WKB	vegetables	AFR
Chillies, BL	vegetables	AFR
Hommus	legumes	WAS
Dried Broad Beans	legumes	WAS
Foul Medammas, canned	legumes	WAS
Mixed Pickles	vegetables	WAS
Green/mung beans, dried, BL	legumes	ASI
Moong dahl, loose, BL	legumes	ASI
Peanuts in shell, BL	groundnuts	ASI
Chillies, dried, BL	spices	ASI
Mushrooms, dried, BL	vegetables	ASI
White sugar, BL	sugar	CIS
Icing sugar, WKB	sugar	CIS
Sugar lumps, WKB	sugar	CIS
Sweetener, tablets, WKB	sugar	CIS
White sugar, WKB	sugar	GLB
Brown sugar, WKB	sugar	GLB
Sweetener, Well-Known Brand	sugar	LAC
White sugar, Sold Loose, BL	sugar	AFR
Brown sugar cubes-prepacked, WKB	sugar	AFR
Glucose, Powdered, WKB	sugar	AFR
White sugar, loose, BNR	sugar	ASI
Honey, mixed blossoms, WKB	honey	CIS
Honey, mixed blossoms, BL	honey	GLB
Honey, mixed blossoms, WKB (ASI)	honey	ASI

SI.2 Consumption data

We estimated baseline food consumption for the year 2017 by adopting estimates of food availability from the FAO's commodity balances and food balance sheets, and adjusting those for the amount of food wasted at the point of consumption.[1,2] Commodity balances and food balance sheets report on the amount of food that is available for human consumption.[2] They reflect the quantities reaching the consumer, but do not include waste from both edible and inedible parts of the food commodity occurring in the household. As such, the amount of food actually consumed may be lower than the quantity shown in the food balance sheet depending on the degree of losses of edible food in the household, e.g. during storage, in preparation and cooking, as plate-waste, or quantities fed to domestic animals and pets, or thrown away.

We followed the waste-accounting methodology developed by the FAO to account for the amount of food wasted at the household level that was not accounted for in food availability estimates.[1] For each commodity and region, we estimated food consumption by multiplying food availability data with conversion factors (*cf*) that represent the amount of edible food (e.g. after peeling) and with the percentage of food wasted during consumption (*1-wp(cns)*). For roots and tubers, fruits and vegetables, and fish and seafood, we also accounted for the differences in wastage between the proportion that is utilised fresh (pct_{frsh}) and the proportion that utilised in processed form (pct_{prcd}). The equation used for each food commodity and region was:

$$Consumption = Availability \cdot \frac{pct_{frsh}}{100} \cdot cf_{frsh} \cdot \left(1 - \frac{wp\left(cns_{frsh}\right)}{100}\right)$$
$$+ Availability \cdot \frac{pct_{prcd}}{100} \cdot cf_{prcd} \cdot \left(1 - \frac{wp\left(cns_{prcd}\right)}{100}\right)$$

SI Table 3 provides and overview of the parameters used in the calculation, and SI Table 4 provides an overview of the baseline consumption data calculated in that way. The differences across energy intake reflect differences in the prevalence of overweight and obesity across regions.[3]

SI Table 3. Percentage of food wasted during consumption (wp_{cns}), and percentage of processed utilisation (pct_{prcd}). The percentage of fresh utilisation is calculated as 1-pct_{prcd}. Conversion factors to edible portions of foods are provided below the table.

Food group	Item	Region						
		Europe	USA, Canada, Oceania	Indus-trialized Asia	Sub-Saharan Africa	North Africa, West and Central Asia	South and Southeast Asia	Latin America
cereals	wp(cns)	25	27	20	1	12	3	10
roots and tuber	pct_{prcd}	73	73	15	50	19	10	80
	wp(cns)	17	30	10	2	6	3	4
	wp(cns_{prcd})	12	12	12	1	3	5	2
oilseeds and pulses	cns	4	4	4	1	2	1	2
fruits and vegetables	pct_{prcd}	60	60	4	1	50	5	50
	wp(cns)	19	28	15	5	12	7	10
	wp(cns_{prcd})	15	10	8	1	1	1	1
milk and dairy	wp(cns)	7	15	5	0.1	2	1	4
eggs	wp(cns)	8	15	5	1	12	2	4
meat	wp(cns)	11	11	8	2	8	4	6
fish and seafood	pct_{prcd}	*40% for low-income countries, and 96% for all others.*						
	wp(cns)	11	33	8	2	4	2	4
	wp(cns_{prcd})	10	10	7	1	2	1	2

Conversion factors : maize, millet, sorghum: 0.69; wheat, rye, other grains: 0.78; rice: 1; roots: 0.74 (0.9 for industrial processing); nuts and seeds: 0.79; oils: 1; vegetables: 0.8 (0.75 for industrial processing); fruits: 0.8 (0.75 for industrial processing); beef: 0.715; lamb: 0.71; pork: 0.68; poultry: 0.71; other meat: 0.7; milk and dairy: 1; fish and seafood: 0.5; other crops: 0.78

SI Table 4. Overview of baseline consumption data by region and food group (in grams per day for each food group, and in kcal per day for total energy intake).

Food group	Global	High-income countries	Upper middle-income countries	Lower middle-income countries	Low-income countries
wheat	116	140	127	108	48
rice	206	53	195	292	122
maize	33	15	31	36	81
other grains	19	10	7	21	91
roots	129	106	122	123	267
fruits (temperate)	75	74	106	55	30
fruits (tropical)	44	48	57	36	18
fruits (starchy)	32	20	24	38	64
vegetables	272	195	472	150	88
legumes	24	13	17	32	50
treenuts	6	8	6	5	4
groundnuts	5	5	7	3	10
vegetable oil	22	44	23	14	11
palm oil	6	3	6	7	8
sugar	48	80	46	41	23
eggs	26	33	46	9	2
milk	152	169	130	178	73
cream	2	6	1	0	0
cheese	7	33	3	1	1
butter	2	7	1	0	0
shellfish	7	11	12	2	0
fish (freshwater)	11	5	15	11	5
fish (pelagic)	4	7	3	4	1
fish (demersal)	4	9	4	2	1
fish (other)	2	1	2	1	2
beef	18	39	21	8	11
lamb	5	5	7	3	6
pork	28	61	38	10	5
poultry	29	55	48	6	3
other crops	70	147	81	27	67
total kcal	2,187	2,222	2,281	2,128	1,939

SI.3 Diet scenarios

For the analysis of healthy and sustainable dietary patterns, we adopted energy-balanced varieties of the flexitarian, pescatarian, vegetarian, and vegan dietary patterns defined by the EAT-Lancet Commission on Healthy Diets from Sustainable Food Systems.[4]

As previously estimated [5] and used by the EAT-Lancet Commission,[4] the average calorie needs differ by region based on its age composition, and range around 2100 kcal/d. For the calculations, which need estimates of healthy body weights (or body mass index, BMI), physical activity levels, and heights as inputs, it was assumed that BMIs are in line with WHO recommendations,[6] and moderately physical activity levels are maintained as recommended. In addition, the US characteristics for height were used,[7] which can be seen as an upper bound that does not penalise future growth of populations. According to the estimates, calorie needs reach a maximum of 2500 kcal/d for ages 20-24 (averaged between men and women), but are reduced to 2000 kcal for ages 65 and older. SI Table 5 provides an overview.

SI Table 5. Calorie needs (kcal/d) by age and sex.

Age	Female	Male	Average
0-4	1200	1200	1200
5-9	1520	1600	1560
10-14	1920	2120	2020
15-19	2040	2760	2400
20-24	2200	2800	2500
25-29	2000	2600	2300
30-34	2000	2600	2300
35-39	2000	2600	2300
40-44	2000	2600	2300
45-49	2000	2400	2200
50-54	1800	2400	2100
55-59	1800	2400	2100
60-64	1800	2400	2000
65-69	1800	2200	2000
70-74	1800	2200	2000
75-79	1800	2200	2000
80-84	1800	2200	2000
85-89	1800	2200	2000
90-94	1800	2200	2000
95-99	1800	2200	2000
100+	1800	2200	2000

The flexitarian diets (FLX) included at least 500 g/d of fruits and vegetables of different colours and groups (the composition of which is determined by regional preferences), at least 100 g/d of plant-based protein sources (legumes, soybeans, nuts), a focus on whole grains, modest amounts of animal-based proteins, such as poultry, fish, milk, and eggs, and limited amounts of red meat (1 portion per week), refined sugar (<5% of total energy), vegetable oils that are high in saturated fat (in particular palm oil), and starchy foods which have a relatively high glycaemic index. SI Table 6 provides an overview of the food-based recommendations used for constructing the flexitarian-diet scenario.

SI Table 6. Food-based dietary recommendations for healthy, more plant-based (flexitarian) diets. The recommendations include recommended minimum (min) and maximum (max) intake expressed by weight or calories, and servings. Fish and seafood can be substituted by plant-based foods (legumes, soybeans, nuts and seeds, fruits and vegetables) in vegetarian diets.

Food item	minimum level		maximum level	
	g/d	serving	g/d	serving
wheat<br>rice<br>maize<br>other grains<br>roots			860 kcal/d for energy balance	3-4 (1/3 of energy)
legumes	50	1/2		
soybeans	25	1/4		
nuts & seeds	50	2		
vegetables	300	3-4		
fruits	200	2-3		
sugar			31	5% of energy
palm oil			6.8	1
vegetable oil			80	1/3 of energy
beef<br>lamb<br>pork			14	1/7
poultry			29	1/2
eggs			13	1/5
milk			250	1
shellfish<br>fish (freshwater)<br>fish (demersal)<br>fish (pelagic)	28	1/2		

Based on the flexitarian diets, we constructed more specialised diets, including pescatarian, vegetarian and vegan diets, which are in line with dietary guidelines and observed dietary patterns in specialised cohorts.[8,9] For the pescatarian diets, meat-based protein sources in the flexitarian diets were replaced (on a kcal basis) to three quarters by fish and seafood, and one quarter by either fruits and vegetables (PSC_{veg}) or whole grains (PSC_{grn}); for the vegetarian diets, they were replaced to three quarters by plant-based proteins, and one quarter by either fruits and vegetables (VEG_{veg}) or whole grains (VEG_{grn}); and for the vegan diets, all animal-based protein sources were replaced to three quarters by plant proteins, and one quarter by either fruits and vegetables (VGN_{veg}) or whole grains (VGN_{grn}). We aimed to preserve the regional character of dietary patterns by maintaining the regional composition of specific foods within broader categories, such as preferences for specific staple crops (wheat, maize, rice, etc) and fruits (temperate, tropical). SI Table 7 provides an overview of the composition of the different diet scenarios.

SI Table 7. Overview of food consumption (g/d for food groups, kcal/d for total) by diet scenarios (net of waste). The diet scenarios include benchmark diets (BMK), flexitarian diets (FLX) and two variants each of pescatarian (PSC), vegetarian (VEG), and vegan (VGN) diets. Each variant is based on the flexitarian diets by replacing animal products with a mix of fish (pescatarian) or legumes (vegetarian, vegan) and either fruits and vegetables (veg) or whole grains (grn).

Food groups	BMK	FLX	PSC^{veg}	PSC^{grn}	VGN^{veg}	VEG^{veg}	VEG^{grn}	VGN^{grn}
wheat	116	73	73	75	73	76	73	82
rice	206	133	133	136	133	138	133	143
maize	33	21	21	22	21	22	21	23
other grains	19	12	12	13	12	13	12	14
roots	129	81	81	81	81	81	81	81
fruits (temperate)	75	117	106	145	106	121	106	106
fruits (tropical)	44	60	67	60	70	60	84	60
fruits (starchy)	32	40	40	40	40	40	40	40
vegetables	272	388	423	388	438	388	505	388
legumes	24	75	75	75	90	90	111	111
treenuts	6	29	29	29	29	29	29	29
groundnuts	5	21	21	21	21	21	21	21
vegetable oil	22	42	42	42	42	42	42	42
palm oil	6	4	4	4	4	4	4	4
sugar	48	31	31	31	31	31	31	31
eggs	26	11	11	11	11	11		
milk	152	128	128	128	128	128		
cream	2	1	1	1	1	1		
cheese	7	6	6	6	6	6		
butter	2	1	1	1	1	1		
shellfish	7	7	21	21				
fish (freshwater)	11	17	38	38				
fish (pelagic)	4	6	14	14				
fish (demersal)	4	5	13	13				
fish (other)	2	2	5	5				
beef	18	5						
lamb	5	2						
pork	29	5						
poultry	28	18						
other crops	70							
total kcal	2,187	2,091	2,091	2,091	2,091	2,091	2,091	2,091

SI.4 Projections of food demand and prices

We used projections of food prices and demand from the International Model for Policy Analysis of Agricultural Commodities and Trade (IMPACT). IMPACT uses economic, water, and crop models to simulate global food production, consumption, and trade of 62 agricultural commodities for over 158 world regions.[10] The IMPACT model system is organized around a core global partial equilibrium multi-market model of agricultural production, demand, trade, and prices. The multi-market model simulates the operation of national and global markets for agricultural commodities, solving for equilibrium prices and quantities. The model specifies supply and demand behaviour in all markets.

For our analysis, we adopted IMPACT's estimates of food demand and consumer prices for different socio-economic pathways that differed in their assumptions on income and population growth, and applied the percentage changes compared to the year 2017 to our baseline price and consumption estimates. The socio-economic scenarios included a "middle-of-the-road" socioeconomic trajectory (SSP2), a "Sustainability"-termed socio-economic pathway (SSP1) which is characterized by medium to high economic growth and low population growth, and a "Fragmentation"-termed socio-economic pathway (SSP3) which is characterized by slow economic growth and high population growth.[11] The scenarios have been specified with GDP projections developed by the Organization for Economic Co-operation and Development (OECD) and population projections developed by the International Institute for Applied Systems Analysis (IIASA).[12,13] SI Table 8 lists the percentage changes in per-capita GDP between 2017 and 2050.

In addition to changes in income and population, the socio-economic pathways take into account changes in consumer preferences, agricultural technology, and policies.[14,15] The "sustainability" pathway with higher income growth includes greater increases in agricultural productivity, stricter land-use regulation, lower barriers to trade, and a trend towards healthier diets with more fruits and vegetables and less meat ; the less optimistic scenario includes changes in the opposite direction. SI Tables 9 and 10 provide an overview of the price and demand projections for the different socio-economic pathways, mapped to the food groups used in the analysis.

SI Table 8. Percentage changes in GDP per person between 2017 and 2050 by socio-economic pathway

Socio-economic scenario	Global	High-income countries	Upper middle-income countries	Lower middle-income countries	Low-income countries
SSP2	114	53	116	225	270
SSP1	179	65	164	348	485
SSP3	57	45	68	123	134

SI Table 9. Percentage changes in commodity prices between 2017 and 2050 by food group, region, and socio-economic pathway

Food group	Global			High-income countries			Upper middle-income countries			Lower middle-income countries			Low-income countries		
	SSP2	SSP1	SSP3	SSP2	SSP1	SSP3	SSP2	SSP1	SSP3	SSP2	SSP1	SSP3	SSP2	SSP1	SSP3
wheat	12	0	26	9	-5	29	11	0	23	12	1	25	12	-1	28
rice	13	-2	36	5	-18	49	14	6	22	17	0	44	14	6	23
maize	30	23	38	29	24	35	28	24	29	30	23	39	30	25	34
other grains	7	-15	34	3	-16	21	3	-14	17	7	-15	36	6	-15	29
roots	14	1	28	7	6	4	10	6	10	14	0	30	15	0	30
fruits (temperate)	12	33	13	9	40	-2	10	39	2	12	30	17	11	34	7
fruits (tropical)	12	29	26	10	36	9	11	34	13	13	26	30	11	30	19
fruits (starchy)	6	23	17	4	31	0	5	28	5	6	20	21	6	22	13
vegetables	29	43	29	26	50	12	27	49	16	29	40	33	27	44	22
legumes	9	2	25	5	7	7	6	6	10	10	-1	32	7	2	18
nuts & seeds	4	-9	20	2	-8	12	3	-6	10	4	-11	25	5	-6	16
sugar	16	3	21	14	-4	27	18	11	10	18	2	27	18	11	10
vegetable oil	11	0	23	8	8	-1	7	5	2	13	-3	32	9	3	11
palm oil	16	9	27	21	22	11	16	17	14	17	5	35	14	12	16
eggs	0	-5	-8	-1	-3	-14	-2	-4	-13	0	-6	-8	0	-6	-8
milk	5	-1	8	4	-1	6	4	-2	6	5	-1	7	5	-1	8
shellfish	7	7	8	11	11	11	6	6	6	8	7	8	4	4	4
fish (freshwater)	8	8	7	9	9	9	9	9	8	7	8	7	8	8	8
fish (pelagic)	35	36	35	21	20	21	37	37	38	40	41	39	36	37	36
fish (demersal)	24	24	23	24	24	24	25	25	25	24	25	24	26	27	26
poultry	5	-33	-8	4	-32	-14	3	-33	-13	5	-34	-8	5	-34	-8
beef	4	-13	0	3	-13	-4	3	-13	-3	4	-13	1	4	-13	0
lamb	-6	-31	-7	-8	-31	-11	-7	-31	-11	-6	-31	-7	-6	-31	-8
pork	8	-35	6	5	-36	-1	8	-34	4	9	-35	7	8	-35	6
other crops	15	10	19	15	17	8	17	17	13	10	1	20	24	24	20

SI Table 10. Percentage changes in food demand between 2017 and 2050 by food group, region, and socio-economic pathway

Food group	Global			High-income countries			Upper middle-income countries			Lower middle-income countries			Low-income countries		
	SSP2	SSP1	SSP3	SSP2	SSP1	SSP3	SSP2	SSP1	SSP3	SSP2	SSP1	SSP3	SSP2	SSP1	SSP3
wheat	4	14	-5	6	10	1	2	8	-4	7	16	-1	13	31	-1
rice	-8	-3	-11	-7	-4	-8	-1	1	-3	-7	-2	-14	-18	-11	-24
maize	9	13	7	0	0	0	0	1	0	12	20	6	5	12	0
other grains	33	43	27	1	5	1	-2	3	-5	26	33	18	40	60	23
roots	9	17	2	-2	-3	-1	-6	-5	-8	10	21	-1	10	20	0
fruits (temperate)	14	50	-1	7	40	9	12	52	7	19	41	2	31	127	10
fruits (tropical)	20	67	4	10	39	10	16	66	10	23	60	6	64	216	24
fruits (starchy)	50	109	31	11	42	12	22	78	15	42	84	22	69	167	35
vegetables	35	85	9	5	39	8	9	55	5	48	97	13	21	118	4
legumes	25	41	14	5	17	4	15	30	9	24	39	9	35	63	16
nuts & seeds	6	13	-2	8	10	6	3	5	2	3	12	-7	20	32	8
sugar	25	13	9	7	-7	5	12	-13	7	43	33	19	32	39	19
vegetable oil	4	2	-6	1	-12	2	10	4	5	8	8	-2	23	31	9
palm oil	60	56	34	19	7	23	41	24	28	66	65	34	58	68	31
eggs	3	10	-6	-2	-2	2	7	7	6	8	15	-3	47	70	23
milk	12	18	-3	2	0	0	6	11	-1	27	35	11	31	44	17
shellfish	3	9	-4	5	6	4	28	27	29	6	11	-1	16	23	9
fish (freshwater)	31	32	30	9	10	8	35	36	32	21	24	17	61	66	57
fish (pelagic)	-26	-23	-29	-20	-19	-19	-13	-14	-13	-16	-16	-16	-53	-54	-52
fish (demersal)	-14	-10	-19	-14	-13	-14	28	27	29	-9	-7	-12	-9	-4	-13
poultry	34	35	13	17	-12	20	28	22	20	63	82	30	73	123	33
beef	23	27	5	6	-4	6	10	4	6	40	52	17	90	125	47
lamb	49	57	28	27	-5	29	38	38	23	42	47	20	107	177	64
pork	-7	-12	-19	0	-14	-1	10	13	1	-10	-20	-20	26	54	-1
other crops	10	21	-2	3	4	3	13	20	6	18	27	9	32	64	10

SI.5 Health analysis and valuation

For estimating the healthcare-related costs of diets, we used a comparative risk assessment of diet and weight-related risk factors, and paired the estimates of cause-specific attributable deaths obtained from that assessment with cost-of-illness estimates.

In the comparative risk assessment, we estimated the mortality and disease burden attributable to dietary and weight-related risk factors by calculating population impact fractions (PIFs) which represent the proportions of disease cases that would be avoided when the risk exposure was changed from a baseline situation to a counterfactual situation. For calculating PIFs, we used the general formula[16–18]:

$$PIF = \frac{\int RR(x)P(x)dx - \int RR(x)P'(x)dx}{\int RR(x)P(x)dx}$$

where $RR(x)$ is the relative risk of disease for risk factor level x, $P(x)$ is the number of people in the population with risk factor level x in the baseline scenario, and $P'(x)$ is the number of people in the population with risk factor level x in the counterfactual scenario. We assumed that changes in relative risks follow a dose-response relationship,[17] and that PIFs combine multiplicatively, i.e. $PIF = 1 - \prod_i (1 - PIF_i)$ where the *i*'s denote independent risk factors.[17,19]

The number of avoided deaths due to the change in risk exposure of risk *i*, $\Delta deaths_i$, was calculated by multiplying the associated PIF by disease-specific death rates, *DR*, and by the number of people alive within a population, *P*:

$$\Delta deaths_i(r,a,d) = PIF_i(r,d) \cdot DR(r,a,d) \cdot P(r,a)$$

where PIFs are differentiated by region *r* and disease/cause of death *d*; the death rates are differentiated by region, age group *a*, and disease; the population groups are differentiated by region and age group; and the change in the number of deaths is differentiated by region, age group and disease.

We used publicly available data sources to parameterize the comparative risk analysis. Mortality data were adopted from the Global Burden of Disease project.[20,21] Baseline data on the weight distribution in each country were adopted from a pooled analysis of population-based measurements undertaken by the NCD Risk Factor Collaboration.[3]

The relative risk estimates that relate the risk factors to the disease endpoints were adopted from meta-analyses of prospective cohort studies for dietary weight-related risks.[22–30] In line with the meta-analyses, we included non-linear dose-response relationships for fruits and vegetables, nuts and seeds, and fish, and assumed linear dose-response relationships for the remaining risk factors. As our analysis was primarily focused on mortality from chronic diseases, we focused on adults aged 20 year or older, and we adjusted the relative-risk estimates for attenuation with age based on a pooled analysis of cohort studies focussed on metabolic risk factors,[31] in line with other assessments.[18,32]

SI Table 11 provides an overview of the relative-risk parameters used. A detailed discussion of the parameter selection can be found elsewhere.[33] For ensuring that the relative risks are well-defined for the whole range of exposures considered in the diet scenarios, we capped the maximum exposure/potential risk reductions at the maximum values included in the meta-analyses (800 g/d of fruits or vegetables, 28 g/d of nuts, 50 g/d of fish). For whole grains, we used a maximum exposure of 125 g/d, in line with the TMREL value suggested by the Global Burden of Disease and the Nutrition and Chronic Diseases Expert Group (NutriCoDE),[32] and we left the linear dose-response functions (for legumes, red meat, and processed meat) unconstraint, but checked that the intake values didn't exceed the values covered in the meta-analyses.

The selection of risk-disease associations used in the health analysis was supported by available criteria used to judge the certainty of evidence, such as the Bradford-Hill criteria used by the Nutrition and Chronic Diseases Expert Group (NutriCoDE),[32] the World-Cancer-Research-Fund criteria used by the Global Burden of Disease project,[34] as well as NutriGrade (SI Table 12).[35] The quality of evidence in meta-analyses that covered the same risk-disease associations as used here was graded with NutriGrade as moderate or high for all risk-disease pairs included in the analysis.[25–27] In addition, the Nutrition and Chronic Diseases Expert Group graded the evidence for a causal association of ten of the 14 cardiometabolic risk associations included in the analysis as probable or convincing,[32] and the World Cancer

SI Table 11. Relative risk parameters (mean and low and high values of 95% confidence intervals) for dietary risks and weight-related risks. We used non-linear dose-response relationships for fruits and vegetables, nuts and seeds, and fish as specified in the references, and we used linear dose-response relationships for the remaining risk factors.

Food group	Endpoint	Unit	RR mean	RR low	RR high	Reference
Processed meat	CHD	50 g/d	1.27	1.09	1.49	Bechthold et al (2019)
	Stroke	50 g/d	1.17	1.02	1.34	Bechthold et al (2019)
	Colorectal cancer	50 g/d	1.17	1.10	1.23	Schwingshackl et al (2018)
	Type 2 diabetes	50 g/d	1.37	1.22	1.55	Schwingshackl et al (2017)
Red meat	CHD	100 g/d	1.15	1.08	1.23	Bechthold et al (2019)
	Stroke	100 g/d	1.12	1.06	1.17	Bechthold et al (2019)
	Colorectal cancer	100 g/d	1.12	1.06	1.19	Schwingshackl et al (2018)
	Type 2 diabetes	100 g/d	1.17	1.08	1.26	Schwingshackl et al (2017)
Fish	CHD	15 g/d	0.94	0.90	0.98	Zheng et al (2012)
Fruits	CHD	100 g/d	0.95	0.92	0.99	Aune et al (2017)
	Stroke	100 g/d	0.77	0.70	0.84	Aune et al (2017)
	Cancer	100 g/d	0.94	0.91	0.97	Aune et al (2017)
Vegetables	CHD	100 g/d	0.84	0.80	0.88	Aune et al (2017)
	Cancer	100 g/d	0.93	0.91	0.95	Aune et al (2017)
Legumes	CHD	57 g/d	0.86	0.78	0.94	Afshin et al (2014)
Nuts	CHD	28 g/d	0.71	0.63	0.80	Aune et al (2016)
Whole grains	CHD	30 g/d	0.87	0.85	0.90	Aune et al (2016b)
	Cancer	30 g/d	0.95	0.93	0.97	Aune et al (2016b)
	Type 2 diabetes	30 g/d	0.65	0.61	0.70	Aune et al (2016b)
Underweight	CHD	15<BMI<18.5	1.17	1.09	1.24	Global BMI Collab (2016)
	Stroke	15<BMI<18.5	1.37	1.23	1.53	Global BMI Collab (2016)
	Cancer	15<BMI<18.5	1.10	1.05	1.16	Global BMI Collab (2016)
	Respiratory disease	15<BMI<18.5	2.73	2.31	3.23	Global BMI Collab (2016)
Overweight	CHD	25<BMI<30	1.34	1.32	1.35	Global BMI Collab (2016)
	Stroke	25<BMI<30	1.11	1.09	1.14	Global BMI Collab (2016)
	Cancer	25<BMI<30	1.10	1.09	1.12	Global BMI Collab (2016)
	Respiratory disease	25<BMI<30	0.90	0.87	0.94	Global BMI Collab (2016)
	Type 2 diabetes	25<BMI<30	1.88	1.56	2.11	Prosp Studies Collab (2009)
Obesity (grade 1)	CHD	30<BMI<35	2.02	1.91	2.13	Global BMI Collab (2016)
	Stroke	30<BMI<35	1.46	1.39	1.54	Global BMI Collab (2016)
	Cancer	30<BMI<35	1.31	1.28	1.34	Global BMI Collab (2016)
	Respiratory disease	30<BMI<35	1.16	1.08	1.24	Global BMI Collab (2016)
	Type 2 diabetes	30<BMI<35	3.53	2.43	4.45	Prosp Studies Collab (2009)
Obesity (grade 2)	CHD	30<BMI<35	2.81	2.63	3.01	Global BMI Collab (2016)
	Stroke	30<BMI<35	2.11	1.93	2.30	Global BMI Collab (2016)
	Cancer	30<BMI<35	1.57	1.50	1.63	Global BMI Collab (2016)
	Respiratory disease	30<BMI<35	1.79	1.60	1.99	Global BMI Collab (2016)
	Type 2 diabetes	30<BMI<35	6.64	3.80	9.39	Prosp Studies Collab (2009)
Obesity (grade 3)	CHD	30<BMI<35	3.81	3.47	4.17	Global BMI Collab (2016)
	Stroke	30<BMI<35	2.33	2.05	2.65	Global BMI Collab (2016)
	Cancer	30<BMI<35	1.96	1.83	2.09	Global BMI Collab (2016)
	Respiratory disease	30<BMI<35	2.85	2.43	3.34	Global BMI Collab (2016)
	Type 2 diabetes	30<BMI<35	12.49	5.92	19.82	Prosp Studies Collab (2009)

Research Fund graded all five of the cancer associations as probable or convincing.[36] The certainty of evidence grading in each case relates to the general relationship between a risk factor and a health outcome, and not to a specific relative-risk value.

We did not include all available risk-disease associations that were graded as having a moderate certainty of evidence and showed statistically significant results in the meta-analyses that included NutriGrade assessments.[25–27] That was because for some associations, such as for milk, more detailed meta-analyses (with more sensitivity analyses) were available that indicated potential confounding with other major dietary risks.[37,38] Such sensitivity analyses were not presented in the meta-analyses that included NutriGrade assessments, but they are important for health assessments that evaluate changes in multiple risk factors.

For the different diet scenarios, we calculated uncertainty intervals associated with changes in mortality based on standard methods of error propagation and the confidence intervals of the relative risk parameters. For the error propagation, we approximated the error distribution of the relative risks by a normal distribution and used that side of deviations from the mean which was largest. This method leads to conservative and potentially larger uncertainty intervals as probabilistic methods, such as Monte Carlo sampling, but it has significant computational advantages, and is justified for the magnitude of errors dealt with here (<50%) (see e.g. IPCC Uncertainty Guidelines).

SI Table 12. Overview of existing ratings on the certainty of evidence for a statistically significant association between a risk factor and a disease endpoint. The ratings include those of the Nutrition and Chronic Diseases Expert Group (NutriCoDE),[32] the World Cancer Research Fund,[36] and NutriGrade.[25–27] The ratings relate to the risk-disease associations in general, and not to the specific relative-risk factor used for those associations in this analysis.

Food group	Endpoint	Association	Certainty of evidence
Fruits	CHD	reduction	NutriCoDE: probable or convincing;<br>NutriGrade: moderate quality of meta-evidence
	Stroke	reduction	NutriCoDE: probable or convincing<br>NutriGrade: moderate quality of meta-evidence
	Cancer	reduction	WCRF: strong evidence (probable) for some cancers<br>NutriGrade: moderate quality of meta-evidence for colorectal cancer
Vegetables	CHD	reduction	NutriCoDE: probable or convincing<br>NutriGrade: moderate quality of meta-evidence
	Cancer	reduction	WCRF: strong evidence (probable) for non-starchy vegetables and some cancers<br>NutriGrade: moderate quality of meta-evidence for colorectal cancer
Legumes	CHD	reduction	NutriCoDE: probable or convincing<br>NutriGrade: moderate quality of meta-evidence
Nuts and seeds	CHD	reduction	NutriCoDE: probable or convincing<br>NutriGrade: moderate quality of meta-evidence
Whole grains	CHD	reduction	NutriCoDE: probable or convincing<br>NutriGrade: moderate quality of meta-evidence
	Cancer	reduction	WCRF: strong evidence (probable) for colorectal cancer<br>NutriGrade: moderate quality of meta-evidence for colorectal cancer
	Type-2 diabetes	reduction	NutriCoDE: probable or convincing<br>NutriGrade: high quality of meta-evidence
Fish	CHD	reduction	NutriCoDE: probable or convincing<br>NutriGrade: moderate quality of meta-evidence
Red meat	CHD	increase	NutriGrade: moderate quality of meta-evidence
	Stroke	increase	NutriGrade: moderate quality of meta-evidence
	Cancer	increase	WCRF: strong evidence (probable) for colorectal cancer<br>NutriGrade: moderate quality of meta-evidence for colorectal cancer
	Type-2 diabetes	increase	NutriCoDE: probable or convincing<br>NutriGrade: high quality of meta-evidence
Processed meat	CHD	increase	NutriCoDE: probable or convincing<br>NutriGrade: moderate quality of meta-evidence
	Stroke	increase	NutriGrade: moderate quality of meta-evidence
	Cancer	increase	WCRF: strong evidence (convincing) for colorectal cancer<br>NutriGrade: moderate quality of meta-evidence for colorectal cancer
	Type-2 diabetes	increase	NutriGrade: high quality of meta-evidence

NutriCoDE: Nutrition and Chronic Diseases Expert Group

NutriGrade: Grading of Recommendations Assessment, Development, and Evaluation (GRADE) tailored to nutrition research

WCRF: World Cancer Research Fund

For valuing the health impacts of diets, we paired cause-specific estimates of attributable deaths in each dietary scenario to a set of cost-of-illness estimates. Cost-of-illness estimates capture both the direct and indirect costs associated with treating a specific disease, including medical and health-care costs (direct), and costs of informal care and from lost working days (indirect) (see e.g. ref [39]). For our calculations, we used a global set of country-specific cost-of-illness estimates adopted from Springmann and colleagues.[40] The dataset is based on detailed cost-of-illness estimates for cardiovascular diseases [39,41] and cancer [42] across the European Union [39,41] [42] which were transferred to other regions and years by scaling the base values by the ratio of health expenditure per capita for direct costs, and by the ratio of GDP per capita (adjusted for purchasing power parity) for indirect costs. The dataset also includes country-specific cost estimates for diabetes [43] that were adjusted for co-morbidities to avoid double-counting of CVD-related complications [44,45]. SI Table 13 provides an overview of the cost-of-illness estimates used in the analysis.

SI Table 13. Healthcare-related costs (USD per case of death) by region, cause of death, and cost component in 2017 and 2050.

Region	Cause	2017					2050				
		total	direct	indirect	indirect (labour)	indirect (care)	total	direct	indirect	indirect (labour)	indirect (care)
Global	CHD	54,103	17,231	36,966	16,071	20,895	127,021	44,258	83,357	36,238	47,118
	Stroke	50,219	24,332	25,906	10,953	14,954	120,473	62,498	58,418	24,698	33,720
	Cancer	61,720	24,156	37,628	26,014	11,615	146,278	62,046	84,851	58,660	26,192
	T2DM	79,156	79,156				183,546	183,546			
HIC	CHD	183,155	74,548	107,817	46,873	60,945	334,851	168,156	166,695	72,469	94,226
	Stroke	181,592	105,270	75,560	31,945	43,615	354,278	237,456	116,822	49,390	67,432
	Cancer	215,203	104,509	109,750	75,874	33,877	405,423	235,740	169,683	117,308	52,377
	T2DM	430,552	430,552				976,117	976,117			
UMC	CHD	62,764	18,028	46,156	20,066	26,090	152,032	56,333	99,443	43,232	56,211
	Stroke	56,902	25,458	32,347	13,676	18,671	146,616	79,548	69,692	29,464	40,227
	Cancer	70,870	25,274	46,983	32,481	14,503	176,388	78,973	101,226	69,981	31,246
	T2DM	56,810	56,810				191,863	191,863			
LMC	CHD	29,671	5,801	23,879	10,381	13,498	101,383	22,351	79,186	34,425	44,760
	Stroke	24,927	8,192	16,735	7,075	9,660	86,924	31,562	55,495	23,462	32,033
	Cancer	32,435	8,133	24,307	16,804	7,503	111,769	31,334	80,605	55,725	24,881
	T2DM	11,246	11,246				41,843	41,843			
LIC	CHD	8,442	1,908	6,594	2,867	3,728	34,432	9,654	24,976	10,858	14,118
	Stroke	7,261	2,695	4,621	1,954	2,668	30,925	13,633	17,503	7,400	10,103
	Cancer	9,319	2,675	6,713	4,641	2,072	38,717	13,534	25,423	17,576	7,848
	T2DM	2,229	2,229				11,137	11,137			

SI.6 Climate-change analysis and valuation

For estimating the climate-change costs of diets, we first calculated the GHG emissions associated with food consumption and then paired those with cost estimates of climate damages.

For the calculation of GHG emissions, we adopted a regionalised, country-level set of emissions factors derived from a comprehensive meta-analysis of life-cycle assessments of food products.[46] The assessments included all main emissions (carbon dioxide, methane, nitrous oxide) and sources along the food supply chain, from deforestation, pesticide and fertilizer production and transport to farm, fertilizer application and related emissions, energy use on farm, animal production and aquaculture emissions, food processing, food packaging, food transport, and food retail. The data has been weighted and resampled to ensure the representativeness of impacts in line with national and global statistics.

We accounted for improvements in the emissions intensities of foods over time by incorporating the mitigation potential of bottom-up changes in management practices and technologies from marginal abatement cost curves,[47] in line with previous assessments.[48] The mitigation options included changes in irrigation, cropping and fertilization that reduce methane and nitrous oxide emissions for rice and other crops, as well as changes in manure management, feed conversion and feed additives that reduce enteric fermentation in livestock. SI Table 14 provides an overview of the emissions footprints used in the analysis.

For monetizing the GHG emissions, we used estimates of the social cost of carbon (SCC) which represents the economic cost caused by an additional ton of GHG emissions. Compared to our earlier study [40], we used estimates from a fully revised version of the Dynamic Integrated model of Climate and the Economy (DICE) for a scenario that constrains future temperature rise (with the temperature limit averaged over 100 y) in line with stated policy goals.[49] The SCC values in that scenario were USD/tCO_2-eq 107, 204, and 543 for the years 2015, 2030, and 2050. An alternative would have been to adopt SCC values obtained for different discount rates (that are used to convert future damages to present values) for a reference path with current policies, or to adopt SCC values for an “optimal control” path, but

neither of these options fulfilled stated policy objectives with respect to limiting climate change.

SI Table 14. GHG emissions footprints (kgCO_2-eq per kg of product) by food commodity and region.

Commodities	2017					2050				
	Global	HIC	UMC	LMC	LIC	Global	HIC	UMC	LMC	LIC
wheat	1.70	1.62	1.62	1.62	2.34	1.58	1.39	1.44	1.49	2.27
rice	3.69	2.86	3.93	3.83	3.94	2.55	1.67	2.48	2.79	2.78
maize	1.33	0.68	1.26	1.27	2.91	1.39	0.59	1.15	1.26	3.06
other grains	1.57	1.61	1.60	1.56	1.47	1.36	1.38	1.44	1.35	1.27
roots	0.57	0.59	0.62	0.50	0.79	0.62	0.59	0.63	0.54	0.87
temperate fruits	0.66	0.64	0.66	0.67	0.64	0.67	0.65	0.67	0.69	0.62
tropical fruits	0.58	0.51	0.83	0.51	0.50	0.58	0.51	0.84	0.51	0.47
starchy fruits	0.72	0.70	0.86	0.67	0.66	0.72	0.70	0.86	0.68	0.66
vegetables	0.78	0.72	0.87	0.72	0.99	0.84	0.73	0.88	0.78	1.13
legumes	2.04	1.83	1.57	2.39	1.79	1.68	1.49	1.43	1.92	1.49
treenuts	1.66	0.04	4.53	1.18	0.58	1.62	0.02	4.67	1.14	0.36
groundnuts	2.39	2.08	3.33	2.12	2.11	2.39	2.08	3.36	2.14	2.12
vegetable oils	6.49	5.00	6.21	6.95	7.49	6.55	4.96	6.31	6.93	7.30
palm oil	7.20	7.69	5.62	7.71	7.32	7.13	7.69	5.52	7.66	7.20
sugar	2.26	1.46	2.98	2.10	2.84	2.40	1.44	3.03	2.20	3.13
beef	48.25	34.91	69.40	44.20	44.93	42.44	29.80	66.23	37.14	37.54
lamb	30.58	22.50	49.64	25.72	26.26	27.67	18.58	47.04	23.20	23.08
pork	7.85	6.83	8.73	7.69	8.53	7.11	5.55	8.26	6.96	7.52
poultry	7.23	5.32	7.00	8.03	7.40	6.33	4.27	6.58	7.05	5.75
eggs	4.51	4.25	4.09	4.75	4.80	3.99	3.45	3.81	4.23	3.99
milk	2.92	2.58	3.66	2.66	3.12	2.67	2.17	3.46	2.39	2.85
cream	2.89	2.58	3.66	2.64	2.96	2.62	2.17	3.46	2.36	2.64
cheese	22.34	18.47	29.42	20.03	24.31	20.62	15.80	27.84	18.03	22.95
butter	2.89	2.58	3.66	2.64	2.96	2.62	2.17	3.46	2.36	2.64
shellfish	10.06	8.20	14.05	9.17	8.92	9.97	8.20	14.30	8.88	8.97
freshwater fish	2.99	1.88	2.53	3.71	2.59	3.02	1.92	2.55	3.76	2.46
pelagic fish	2.99	1.88	2.53	3.71	2.59	3.02	1.92	2.55	3.76	2.46
demersal fish	2.99	1.88	2.53	3.71	2.59	3.02	1.92	2.55	3.76	2.46

SI.7 Supplementary results

SI Figure 1. Prices per serving (top) and calorie (bottom) of food groups in 2017 by world region, including high-income (HIC), upper middle-income (UMC), lower middle-income (LMC), and low-income (LIC) countries, as well as a global average (Avg).

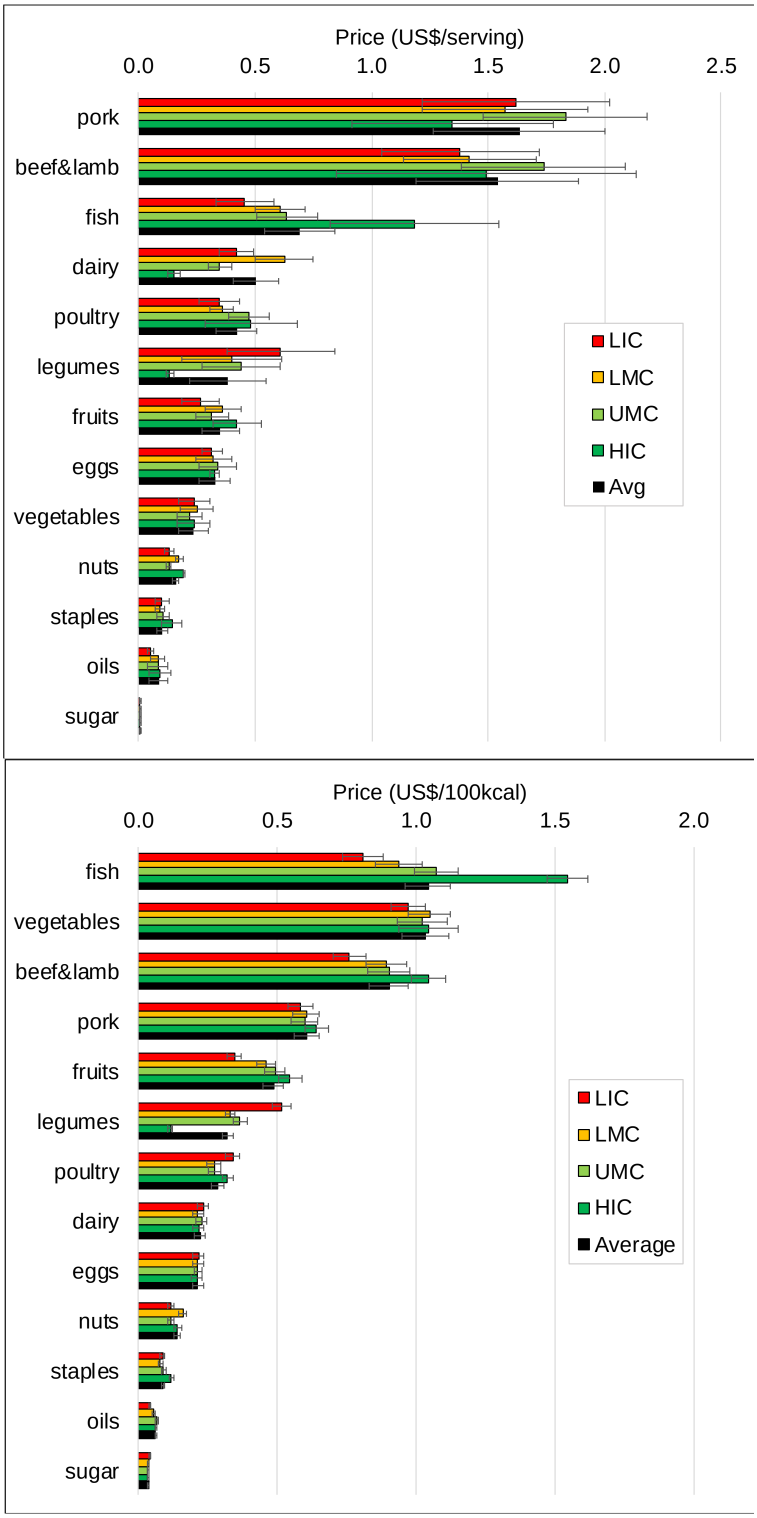

SI Figure 2. Average cost of diets in 2017 differentiated by food consumed and food wasted.

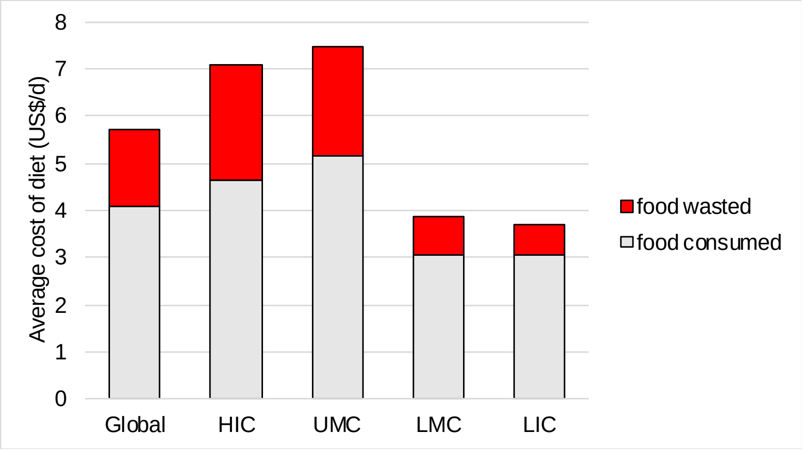

SI Figure 3. Cost of diets in 2017 and 2050 by world region grouped by income, food group, and socio-economic development pathway (med, high, low).

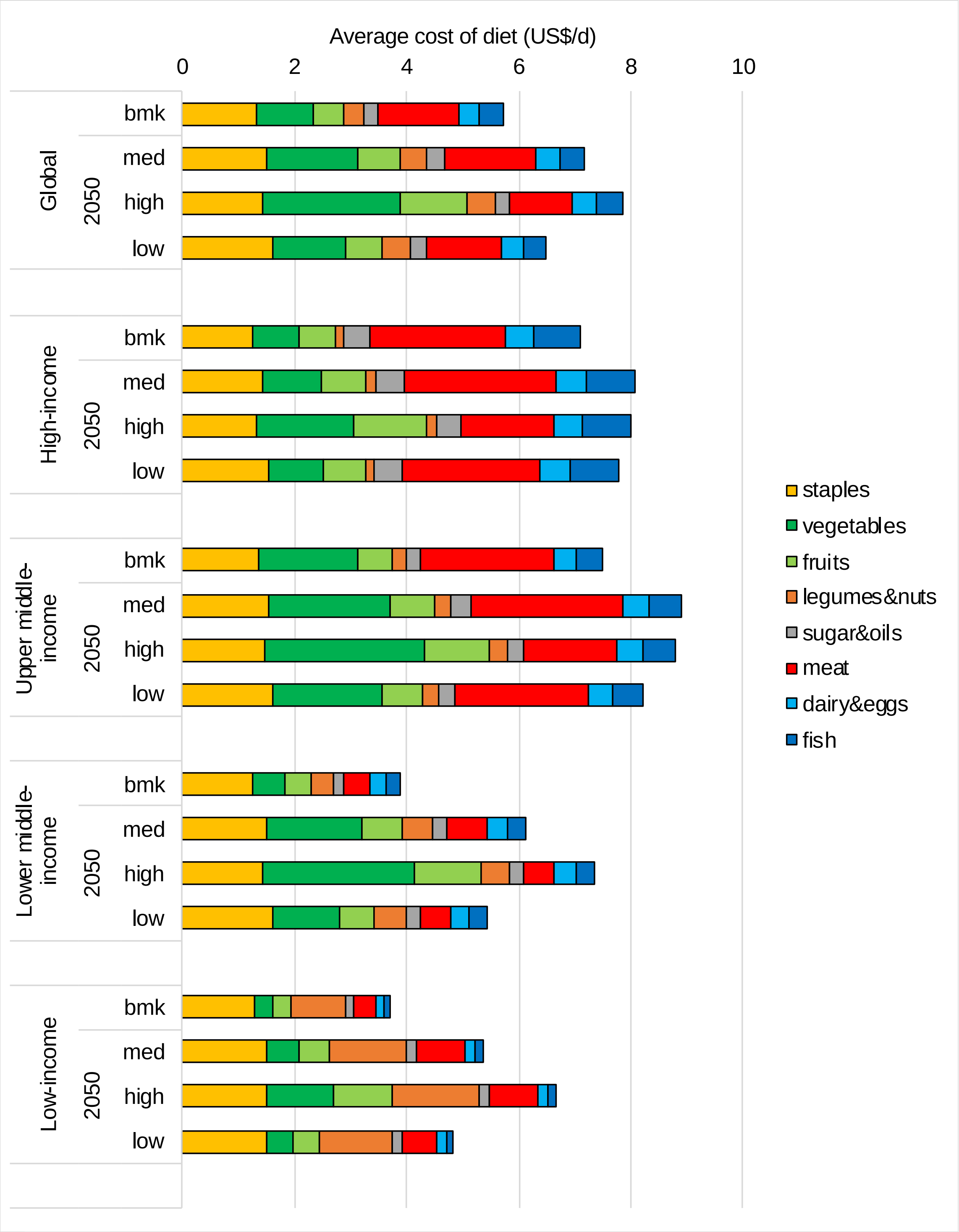

SI Figure 4. Preventable deaths (in thousands) by scenario, cause of deaths, and risk factor. The causes of deaths include coronary heart disease (CHD), stroke, cancer, and type-2 diabetes mellitus (T2DM).

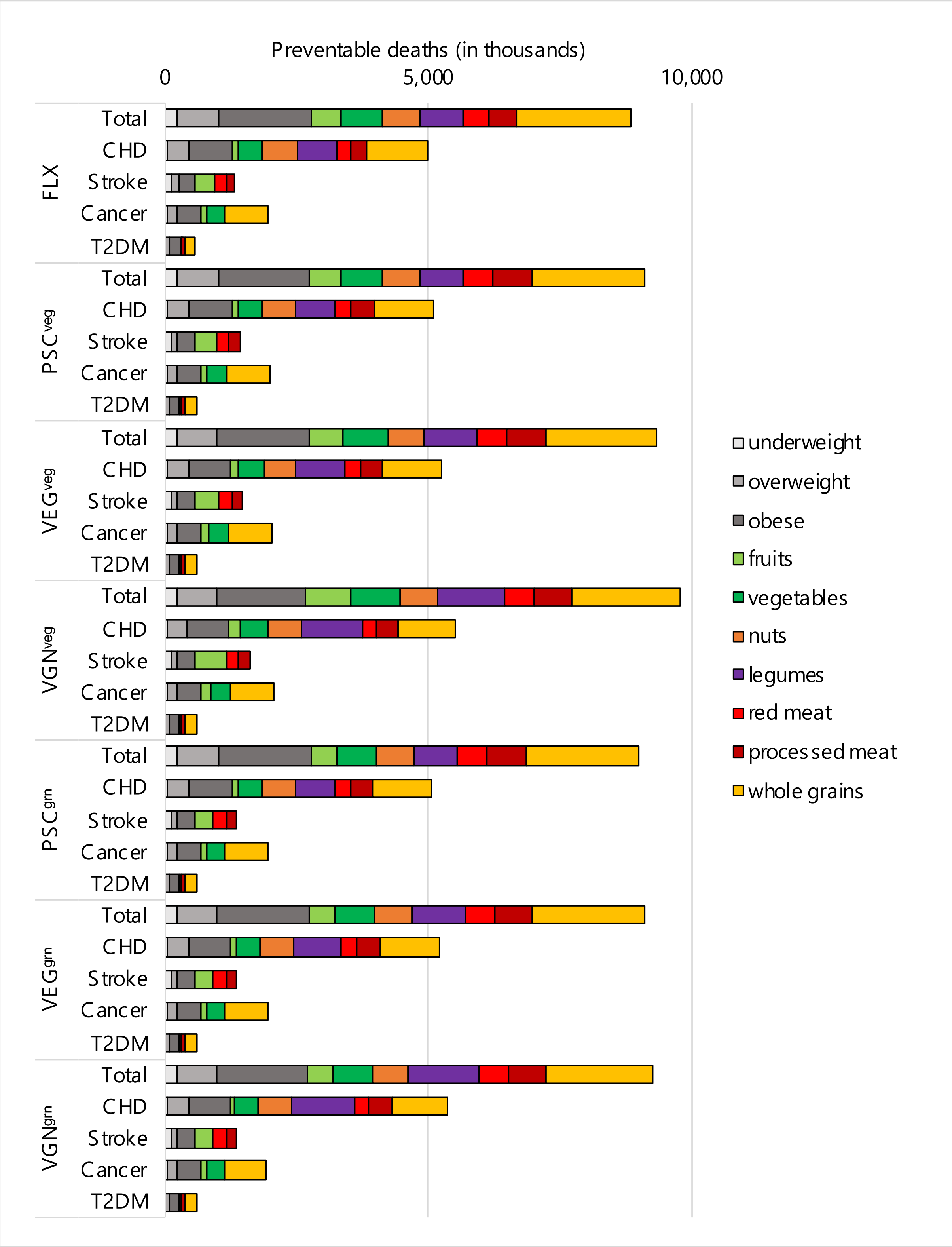

SI Table 15. Healthcare-related costs by world region, scenario, year and socio-economic pathway.

Region	Scenario	Total costs (US$ billion per year)				Costs per person (US$ per year)			
		2017 (bmk)	2050 (med)	2050 (high)	2050 (low)	2017 (bmk)	2050 (med)	2050 (high)	2050 (low)
Global	BMK	662.5	2,038.4	1,858.3	2,845.2	91.1	223.1	220.5	286.6
	PSC^{veg}	91.4	204.9	181.6	283.8	12.6	22.4	21.5	28.6
	PSC^{gm}	79.4	165.1	150.1	264.9	10.9	18.1	17.8	26.7
	FLX	67.5	129.1	119.0	227.9	9.3	14.1	14.1	23.0
	VGN^{veg}	47.8	82.2	82.2	174.1	6.6	9.0	9.8	17.5
	VEG^{veg}	83.2	178.7	175.6	271.2	11.4	19.6	20.8	27.3
	VEG^{gm}	76.9	161.4	162.8	248.9	10.6	17.7	19.3	25.1
	VGN^{gm}	67.4	133.9	141.4	214.5	9.3	14.7	16.8	21.6
High-income	BMK	410.7	1,021.0	927.2	1,601.3	376.2	811.6	722.6	1,494.0
	PSC^{veg}	60.7	64.0	46.4	153.5	55.6	50.9	36.2	143.3
	PSC^{gm}	58.0	53.3	43.1	156.0	53.1	42.3	33.6	145.5
	FLX	51.5	39.8	32.7	140.0	47.1	31.6	25.5	130.6
	VGN^{veg}	45.0	35.5	36.1	129.7	41.2	28.2	28.1	121.0
	VEG^{veg}	56.4	55.7	46.5	150.9	51.7	44.3	36.2	140.8
	VEG^{gm}	53.3	49.3	41.9	142.8	48.8	39.2	32.7	133.2
	VGN^{gm}	47.5	37.8	33.8	128.2	43.5	30.0	26.3	119.7
Upper middle-income	BMK	118.4	447.9	299.7	478.8	117.1	388.3	279.2	380.5
	PSC^{veg}	9.0	49.3	31.2	44.0	8.9	42.8	29.0	35.0
	PSC^{gm}	6.9	44.8	30.7	42.0	6.9	38.8	28.5	33.4
	FLX	6.3	42.0	27.8	38.7	6.2	36.4	25.9	30.7
	VGN^{veg}	1.6	34.8	25.8	29.5	1.6	30.2	24.1	23.5
	VEG^{veg}	7.4	43.9	30.1	41.7	7.3	38.1	28.1	33.1
	VGN^{gm}	6.3	41.4	28.6	38.7	6.2	35.9	26.6	30.7
	VEG^{gm}	4.4	36.9	25.7	33.4	4.3	32.0	23.9	26.6
Lower middle-income	BMK	128.8	532.3	584.4	721.1	31.6	106.9	128.3	129.7
	PSC^{veg}	21.4	87.4	100.8	81.2	5.2	17.5	22.1	14.6
	PSC^{gm}	14.1	63.5	73.6	62.6	3.4	12.7	16.2	11.3
	VGN^{veg}	9.5	45.0	56.8	45.7	2.3	9.0	12.5	8.2
	FLX	1.1	9.8	19.5	12.3	0.3	2.0	4.3	2.2
	VEG^{veg}	19.2	75.6	96.2	73.9	4.7	15.2	21.1	13.3
	VGN^{gm}	17.1	67.7	89.8	63.2	4.2	13.6	19.7	11.4
	VEG^{gm}	15.4	56.6	79.8	48.9	3.8	11.4	17.5	8.8
Low-income	BMK	4.7	37.3	46.7	43.9	4.4	22.0	31.6	22.2
	PSC^{veg}	0.3	4.2	3.1	5.0	0.3	2.5	2.1	2.6
	PSC^{gm}	0.4	3.5	2.7	4.3	0.4	2.1	1.8	2.2
	VGN^{veg}	0.2	2.3	1.7	3.6	0.2	1.3	1.1	1.8
	FLX	0.0	2.0	0.8	2.6	0.0	1.2	0.6	1.3
	VEG^{veg}	0.2	3.4	2.8	4.7	0.2	2.0	1.9	2.4
	VGN^{gm}	0.2	3.0	2.5	4.3	0.2	1.8	1.7	2.2
	VEG^{gm}	0.2	2.6	2.1	3.9	0.1	1.5	1.4	2.0

SI Figure 5. Food-related GHG emissions by scenario, food group, and year.

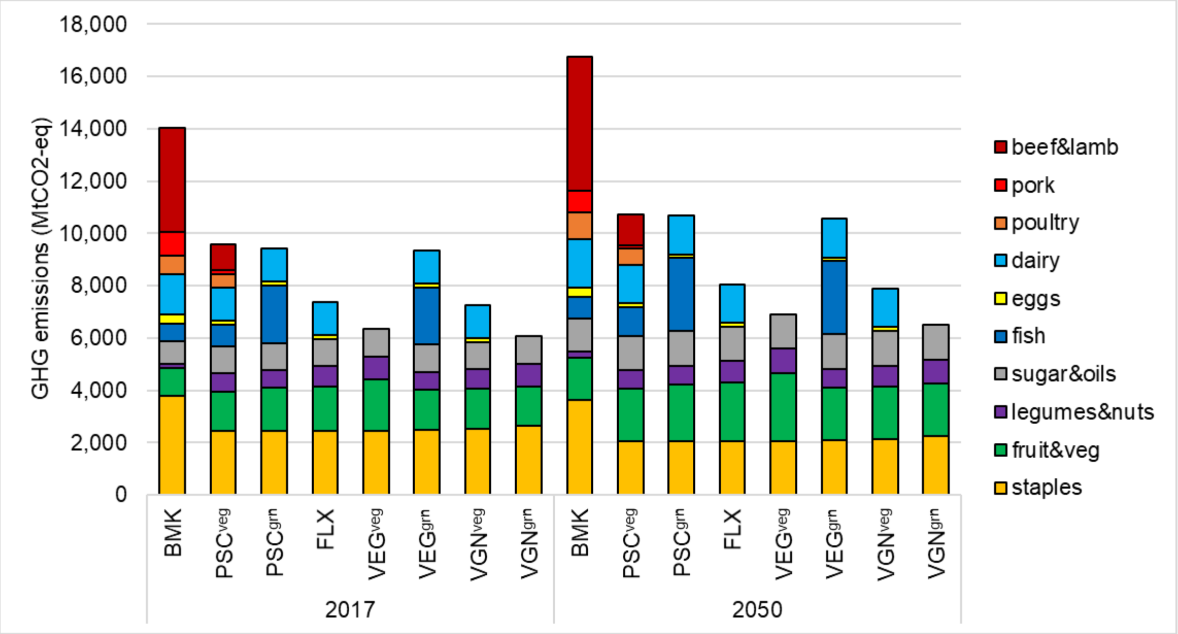

SI Table 16. Social cost of climate change mitigation by world region, scenario, year and socio-economic pathway.

Region	Scenario	Total costs (US$ billion per year)				Costs per person (US$ per year)			
		2017 (bmk)	2050 (med)	2050 (high)	2050 (low)	2017 (bmk)	2050 (med)	2050 (high)	2050 (low)
Global	BMK	1,819	9,295	9,140	9,106	255	1,091	1,149	985
	PSC^{veg}	1,218	5,836	5,620	6,192	171	685	706	670
	PSC^{grn}	1,201	5,798	5,594	6,133	168	680	703	663
	FLX	939	4,373	4,210	4,662	132	513	529	504
	VGN^{veg}	807	3,757	3,626	4,027	113	441	456	436
	VEG^{veg}	1,191	5,735	5,530	6,069	167	673	695	656
	VEG^{grn}	925	4,283	4,122	4,570	130	503	518	494
	VGN^{grn}	773	3,530	3,403	3,797	108	414	428	411
High-income	BMK	336	1,467	1,422	1,244	309	1,186	1,118	1,182
	PSC^{veg}	183	790	821	671	168	638	645	637
	PSC^{grn}	179	818	849	693	165	661	667	658
	FLX	141	609	635	517	130	493	499	491
	VGN^{veg}	103	459	482	391	95	371	379	371
	VEG^{veg}	178	809	839	685	164	654	660	650
	VEG^{grn}	139	597	622	506	128	482	489	481
	VGN^{grn}	96	423	444	360	89	342	349	342
Upper middle-income	BMK	410	2,021	1,847	2,124	420	1,858	1,812	1,796
	PSC^{veg}	202	909	863	992	206	836	846	839
	PSC^{grn}	174	806	772	877	179	741	757	742
	FLX	146	651	627	706	149	599	616	597
	VGN^{veg}	114	510	495	552	117	469	485	467
	VEG^{veg}	172	795	761	865	176	731	746	731
	VGN^{grn}	143	636	613	690	146	585	602	583
	VEG^{grn}	106	468	455	507	109	431	447	429
Lower middle-income	BMK	885	4,493	4,471	4,434	213	935	1,010	821
	PSC^{veg}	684	3,238	3,120	3,495	165	674	705	647
	PSC^{grn}	713	3,366	3,237	3,635	172	700	731	673
	VGN^{veg}	532	2,414	2,314	2,631	128	502	523	487
	FLX	477	2,143	2,066	2,331	115	446	467	432
	VEG^{veg}	708	3,333	3,202	3,600	171	693	723	667
	VGN^{grn}	525	2,365	2,265	2,580	127	492	512	478
	VEG^{grn}	460	2,022	1,948	2,206	111	421	440	409
Low-income	BMK	184	1,250	1,335	1,244	204	933	1,116	802
	PSC^{veg}	147	863	782	992	163	644	653	640
	PSC^{grn}	131	778	708	895	145	581	592	577
	VGN^{veg}	118	671	607	776	130	501	507	501
	FLX	111	625	564	729	123	467	471	470
	VEG^{veg}	130	770	700	886	144	575	585	571
	VGN^{grn}	116	658	595	763	129	491	497	492
	VEG^{grn}	108	599	538	701	120	447	450	452

SI Table 17. Positive and negative changes in cost summed over all countries.

Diet and food-system changes	Number of ountries with cost reductions	Percent of ountries with cost reductions	Population of countries with cost reductions	Number of countries with cost increases	Percent of countries with cost increases	Population of countries with cost increases
In 2017:						
PSC^{veg}	24	16%	2,638,500	126	84%	4,496,720
PSC^{grn}	33	22%	2,814,590	117	78%	4,320,620
FLX	54	36%	3,233,660	96	64%	3,901,560
VGN^{veg}	61	41%	3,439,260	89	59%	3,695,950
VEG^{veg}	72	48%	3,611,140	78	52%	3,524,070
VEG^{grn}	81	54%	3,857,560	69	46%	3,277,660
VGN^{grn}	81	54%	3,804,250	69	46%	3,330,960
in 2017, with halving of waste:						
PSC^{veg}	61	41%	3,446,590	89	59%	3,688,620
PSC^{grn}	67	45%	3,541,020	83	55%	3,594,200
VGN^{veg}	78	52%	3,779,100	72	48%	3,356,110
FLX	78	52%	3,827,430	72	48%	3,307,780
VEG^{veg}	89	59%	3,987,390	61	41%	3,147,820
VEG^{grn}	101	67%	4,117,330	49	33%	3,017,880
VGN^{grn}	97	65%	4,073,820	53	35%	3,061,390
in 2030, with halving of waste, and positive development:						
PSC^{veg}	69	46%	3,622,370	81	54%	3,924,760
PSC^{grn}	79	53%	5,190,130	71	47%	2,357,000
VGN^{veg}	90	60%	4,023,110	60	40%	3,524,020
FLX	98	65%	5,662,940	52	35%	1,884,190
VEG^{veg}	109	73%	6,232,090	41	27%	1,315,040
VEG^{grn}	118	79%	6,614,470	32	21%	932,660
VGN^{grn}	116	77%	6,602,610	34	23%	944,524
in 2030, with halving of waste, and positive development, and fuller cost accounting:						
PSC^{veg}	102	68%	5,839,890	48	32%	1,707,240
PSC^{grn}	111	74%	6,229,270	39	26%	1,317,860
VGN^{veg}	115	77%	6,560,660	35	23%	986,467
FLX	116	77%	6,330,470	34	23%	1,216,660
VEG^{veg}	126	84%	6,890,380	24	16%	656,749
VEG^{grn}	129	86%	6,928,450	21	14%	618,676
VGN^{grn}	129	86%	6,873,750	21	14%	673,379
in 2050, with halving of waste, and positive development, and fuller cost accounting:						
PSC^{veg}	134	89%	7,606,260	16	11%	348,961
PSC^{grn}	136	91%	7,608,830	14	9%	346,390
VGN^{veg}	140	93%	7,667,550	10	7%	287,662
FLX	142	95%	7,706,460	8	5%	248,757
VEG^{veg}	144	96%	7,775,490	6	4%	179,729
VEG^{grn}	145	97%	7,786,370	5	3%	168,847
VGN^{grn}	144	96%	7,775,490	6	4%	179,729

SI Figure 6. Diet costs for baseline diets (BMK), high-income Western diets (HIC), and flexitarian diets (FLX) in 2017 (top), 2030 (middle), and 2050 (low) by cost component and region. The cost components include the market prices, health-related costs, and climate-change costs. The high-income diet is defined in SI Table 4, and the others in SI Table 7.

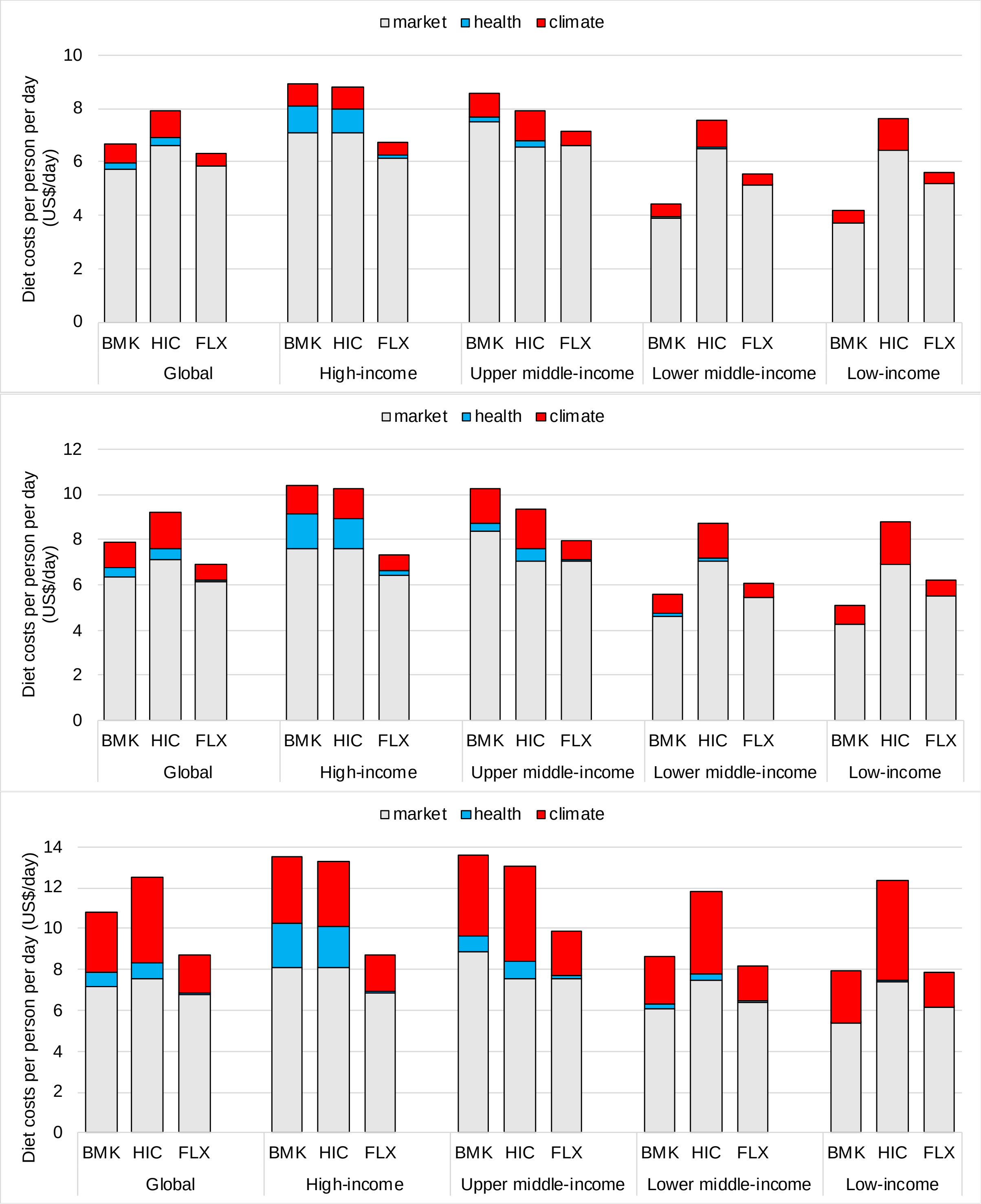

References

1 Gustavsson J, Cederberg C, Sonesson U, Van Otterdijk R, Meybeck A. Global food losses and food waste: extent, causes and prevention. FAO Rome, 2011.

2 Food and Agriculture Organization of the United Nations. Food balance sheets: a handbook. Rome, 2001.

3 NCD Risk Factor Collaboration (NCD-RisC). Trends in adult body-mass index in 200 countries from 1975 to 2014: a pooled analysis of 1698 population-based measurement studies with 19·2 million participants. *Lancet* 2016; **387**: 1377–96.

4 Willett W, Rockström J, Loken B, *et al.* Food in the Anthropocene: the EAT-Lancet Commission on healthy diets from sustainable food systems. *Lancet (London, England)* 2019; **393**: 447–92.

5 Springmann M, Wiebe K, Mason-D'Croz D, Sulser TB, Rayner M, Scarborough P. Health and nutritional aspects of sustainable diet strategies and their relationship to environmental impacts – a comparative global modelling analysis with country-level detail. *Lancet Planet Heal* 2018; **2**: e451–61.

6 World Health Organization. Human energy requirements: Report of a Joint FAO/WHO/UNU Expert Consultation, Rome, Italy, 17-24 October 2001. Geneva, Switzerland: WHO, 2004.

7 Health USD of, Services H. Dietary guidelines for Americans 2015-2020. Skyhorse Publishing Inc., 2017.

8 Haddad EH, Tanzman JS. What do vegetarians in the United States eat? *Am J Clin Nutr* 2003; **78**: 626S-632S.

9 Bradbury KE, Tong TYN, Key TJ. Dietary Intake of High-Protein Foods and Other Major Foods in Meat-Eaters, Poultry-Eaters, Fish-Eaters, Vegetarians, and Vegans in UK Biobank. *Nutrients* 2017; **9**. DOI:10.3390/nu9121317.

10 Robinson S, Mason-D'Croz D, Islam S, *et al.* The International Model for Policy Analysis of Agricultural Commodities and Trade (IMPACT) -- Model description for version 3. 2015.

11 O'Neill BC, Carter T, Ebi KL, *et al.* Meeting Report of the Workshop on The Nature and Use of New Socioeconomic Pathways for Climate Change Research. 2012; published online Jan 17.

12 KC S, Lutz W. The human core of the shared socioeconomic pathways: Population scenarios by age, sex, and level of education for all countries to 2100. *Glob Environ Chang* 2014.

13 Chateau J, Dellink R, Lanzi E, Magne B. Long-term economic growth and environmental pressure: reference scenarios for future global projections. OECD. 2012.

14 Mason-D'Croz D, Bogard JR, Sulser TB, *et al.* Gaps between fruit and vegetable production, demand, and recommended consumption at global and national levels: an integrated modelling study. *Lancet Planet Heal* 2019; **3**: e318–29.

15 Stehfest E, van Zeist WJ, Valin H, *et al.* Key determinants of global land-use projections. *Nat Commun* 2019; **10**. DOI:10.1038/s41467-019-09945-w.

16 Murray CJL, Ezzati M, Lopez AD, Rodgers A, Vander Hoorn S. Comparative quantification of health risks: conceptual framework and methodological issues. *Popul Health Metr* 2003; **1**: 1.

17 Lim SS, Vos T, Flaxman AD, *et al.* A comparative risk assessment of burden of disease and injury attributable to 67 risk factors and risk factor clusters in 21 regions, 1990–2010: a systematic analysis for the Global Burden of Disease Study 2010. *Lancet* 2012; **380**: 2224–60.

18 Forouzanfar MH, Alexander L, Anderson HR, *et al.* Global, regional, and national comparative risk assessment of 79 behavioural, environmental and occupational, and metabolic risks or clusters of risks in 188 countries, 1990–2013: a systematic analysis for the Global Burden of Disease Study 2013. *Lancet* 2015; **386**: 2287–323.

19 Murray CJL, Ezzati M, Flaxman AD, *et al.* GBD 2010: design, definitions, and metrics. *Lancet* 2012; **380**: 2063–6.

20 Lozano R, Naghavi M, Foreman K, *et al.* Global and regional mortality from 235 causes of death for 20 age groups in 1990 and 2010: a systematic analysis for the Global Burden of Disease Study 2010. *Lancet* 2012; **380**: 2095–128.

21 Foreman KJ, Marquez N, Dolgert A, *et al.* Forecasting life expectancy, years of life lost, and all-cause and cause-specific mortality for 250 causes of death: reference and alternative scenarios for 2016–40 for 195 countries and territories. *Lancet* 2018; **392**: 2052–90.

22 Afshin A, Micha R, Khatibzadeh S, Mozaffarian D. Consumption of nuts and legumes and risk of incident ischemic heart disease, stroke, and diabetes: a systematic review and meta-analysis. *Am J Clin Nutr* 2014; : ajcn.076901.

23 Aune D, Keum N, Giovannucci E, *et al.* Nut consumption and risk of cardiovascular disease, total cancer, all-cause and cause-specific mortality: a systematic review and dose-response meta-analysis of prospective studies. *BMC Med* 2016; **14**: 207.

24 Aune D, Giovannucci E, Boffetta P, *et al.* Fruit and vegetable intake and the risk of cardiovascular disease, total cancer and all-cause mortality–a systematic review and dose-response meta-analysis of prospective studies. *Int J Epidemiol* 2016; published online March 18.

25 Bechthold A, Boeing H, Schwedhelm C, *et al.* Food groups and risk of coronary heart disease, stroke and heart failure: A systematic review and dose-response meta-analysis of prospective studies. *Crit Rev Food Sci Nutr* 2019; **59**: 1071–90.

26 Schwingshackl L, Hoffmann G, Lampousi AM, *et al.* Food groups and risk of type 2 diabetes mellitus: a systematic review and meta-analysis of prospective studies. *Eur J Epidemiol* 2017; **32**: 363–75.

27 Schwingshackl L, Schwedhelm C, Hoffmann G, *et al.* Food groups and risk of colorectal cancer. *Int J Cancer* 2018; **142**: 1748–58.

28 Zheng J, Huang T, Yu Y, Hu X, Yang B, Li D. Fish consumption and CHD mortality: an updated meta-analysis of seventeen cohort studies. *Public Health Nutr* 2012; **15**: 725–37.

29 Global BMI Mortality Collaboration E Di, Di Angelantonio E, Bhupathiraju S, *et al.* Body-mass index and all-cause mortality: individual-participant-data meta-analysis of 239 prospective studies in four continents. *Lancet (London, England)* 2016; **388**: 776–86.

30 Aune D, Keum N, Giovannucci E, *et al.* Whole grain consumption and risk of

cardiovascular disease, cancer, and all cause and cause specific mortality: systematic review and dose-response meta-analysis of prospective studies. *BMJ* 2016; **353**: i2716.

31 Singh GM, Danaei G, Farzadfar F, *et al.* The Age-Specific Quantitative Effects of Metabolic Risk Factors on Cardiovascular Diseases and Diabetes: A Pooled Analysis. *PLoS One* 2013; **8**: e65174.

32 Micha R, Shulkin ML, Peñalvo JL, *et al.* Etiologic effects and optimal intakes of foods and nutrients for risk of cardiovascular diseases and diabetes: Systematic reviews and meta-analyses from the Nutrition and Chronic Diseases Expert Group (NutriCoDE). *PLoS One* 2017; **12**: e0175149.

33 Springmann M, Spajic L, Clark MA, *et al.* The healthiness and sustainability of national and global food based dietary guidelines: modelling study. *BMJ* 2020; **370**: m2322.

34 GBD 2017 Diet Collaborators, Afshin A, Sur PJ, Fay KA, *et al.* Health effects of dietary risks in 195 countries, 1990-2017: a systematic analysis for the Global Burden of Disease Study 2017. *Lancet (London, England)* 2019; **0**. DOI:10.1016/S0140-6736(19)30041-8.

35 Schwingshackl L, Knüppel S, Schwedhelm C, *et al.* Perspective: NutriGrade: A Scoring System to Assess and Judge the Meta-Evidence of Randomized Controlled Trials and Cohort Studies in Nutrition Research. *Adv Nutr An Int Rev J* 2016; **7**: 994–1004.

36 World Cancer Research Fund/American Institute for Cancer Research. Diet, Nutrition, Physical Activity and Cancer: A Global Perspective. Continuous Update Project Expert Report. 2018.

37 Aune D, Norat T, Romundstad P, Vatten LJ. Dairy products and the risk of type 2 diabetes: a systematic review and dose-response meta-analysis of cohort studies. *Am J Clin Nutr* 2013; **98**: 1066–83.

38 Aune D, Lau R, Chan DSM, *et al.* Dairy products and colorectal cancer risk: a systematic review and meta-analysis of cohort studies. *Ann Oncol Off J Eur Soc Med Oncol* 2012; **23**: 37–45.

39 Leal J, Luengo-Fernández R, Gray A, Petersen S, Rayner M. Economic burden of

cardiovascular diseases in the enlarged European Union. *Eur Heart J* 2006; **27**: 1610–9.

40 Springmann M, Godfray HCJ, Rayner M, Scarborough P. Analysis and valuation of the health and climate change cobenefits of dietary change. *Proc Natl Acad Sci* 2016; **113**: 4146–51.

41 Nichols M, Townsend N, Scarborough P, Rayner M. European cardiovascular disease statistics. 2012.

42 Luengo-Fernandez R, Leal J, Gray A, Sullivan R. Economic burden of cancer across the European Union: a population-based cost analysis. *Lancet Oncol* 2013; **14**: 1165–74.

43 Zhang P, Zhang X, Brown J, *et al.* Global healthcare expenditure on diabetes for 2010 and 2030. *Diabetes Res Clin Pract* 2010; **87**: 293–301.

44 American Diabetes Association. Economic costs of diabetes in the US in 2012. *Diabetes Care* 2013; **36**: 1033–46.

45 Köster I, Huppertz E, Hauner H, Schubert I. Direct costs of diabetes mellitus in Germany-CoDiM 2000-2007. *Exp Clin Endocrinol diabetes Off journal, Ger Soc Endocrinol [and] Ger Diabetes Assoc* 2011; **119**: 377–85.

46 Poore J, Nemecek T. Reducing food’s environmental impacts through producers and consumers. *Science* 2018; **360**: 987–92.

47 Beach RH, Creason J, Ohrel SB, *et al.* Global mitigation potential and costs of reducing agricultural non-CO2 greenhouse gas emissions through 2030. *J Integr Environ Sci* 2015; **12**: 87–105.

48 Springmann M, Clark M, Mason-D’Croz D, *et al.* Options for keeping the food system within environmental limits. *Nature* 2018; **562**: 519–25.

49 Nordhaus WD. Revisiting the social cost of carbon. *Proc Natl Acad Sci U S A* 2017; **114**: 1518–23.
