# Supplementary material for: The global and regional costs of healthy and sustainable dietary patterns: a modelling study
Source: Lancet Planet Health. 2021 Oct 27;5(11):e797–807. doi: 10.1016/S2542-5196(21)00251-5 (PMC8581186; doi:10.1016/S2542-5196(21)00251-5)
Supplement: Supplementary appendix 2 [file mmc2.pdf]

THE LANCET
Oncology

Supplementary appendix 2

This appendix formed part of the original submission and has been peer reviewed. We post it as supplied by the authors.

Supplement to: Springmann M, Clark MA, Rayner M, Scarborough P, Webb P. The global and regional costs of healthy and sustainable dietary patterns: a modelling study. *Lancet Oncol* 2021; published online Oct 26. http://dx.doi.org/10.1016/S2542-5196(21)00251-5.
